# Response-Surface-Guided Extraction and Copper Modification of Nanocellulose from Corn Cob and Hemp Hurds: Source- and Loading-Dependent Structural and Electrochemical Properties

**DOI:** 10.3390/polym18202547

**Published:** 2026-10-16

**Authors:** Zahra Ranjbar-Navazi

**Affiliations:** Department of Materials Science and Nanotechnology Engineering, Faculty of Engineering, Yeditepe University, 34755 Istanbul, Türkiye; zahra.navazi@yeditepe.edu.tr

**Keywords:** nanocellulose, biomass, corn cob, hemp hurds, response surface methodology, copper modification, electrochemical properties

## Abstract

Agricultural residues offer a source of nanocellulose for developing functional composites. This study examined how biomass source, extraction conditions, and copper loading affect the structural and electrochemical properties of nanocellulose derived from hemp hurds and corn cob. A citric-acid-assisted chemical–mechanical extraction route was investigated using response surface methodology, with extraction cycle number and sonication time as factors and FTIR-derived total crystallinity index and SEM-derived apparent fiber size as responses. The selected conditions were three cycles and 120 min for hemp hurds and three cycles and 75 min for corn cob, yielding measured mean apparent fiber sizes of 83.3 and 100 nm, respectively. Nanocellulose was modified with nominal Cu loadings of 15, 25, 50, and 75 wt%. At 0.1 Hz, impedance magnitude decreased from 274.15 and 247.67 kΩ for the respective bare references to below 10 kΩ for all Cu-modified groups. Cu incorporation also increased cycle-5 passed charge, with the corn-cob series showing its highest mean response at 50 wt%. FTIR, SEM, TGA, and XRD supported cellulose enrichment, nanoscale fibrillation, source-dependent thermal behavior, and crystalline metallic Cu formation. These findings support further evaluation of Cu-modified nanocellulose for electrochemical applications.

## 1. Introduction

Nanocellulose has attracted considerable attention as a renewable nanomaterial because of its low density, high specific surface area, favorable mechanical properties, abundant surface hydroxyl groups, and ability to form interconnected fibrillar networks. Depending on the biomass source and processing route, cellulose nanomaterials can differ substantially in dimensions, structural organization, surface chemistry, and interfacial behavior, which directly influence their suitability for applications in composites, membranes, sensors, and energy-related systems [1,2,3]. The utilization of lignocellulosic residues as feedstocks is particularly attractive because it combines nanomaterial production with biomass valorization and the conversion of agricultural wastes into value-added functional materials.

The characteristics of nanocellulose are strongly affected by both the botanical source and the extraction history. Differences in cellulose organization, lignin and hemicellulose content, ash, extractives, and native fiber architecture can influence chemical purification, fibrillation efficiency, and the structural properties of the resulting nanocellulose [1,2,3]. Corn cob and hemp hurds are abundant agricultural residues with distinctly different chemical compositions and fiber morphologies. Nanocellulose has previously been produced from corn-cob-derived biomass through combined chemical and mechanical treatments [4], while hemp-derived residues have also been investigated as sustainable cellulose sources [5,6]. However, direct comparison of these two biomass sources under an identical extraction and optimization framework remains limited.

Hemp-derived cellulose has also been investigated as a feedstock for regenerated textile fibers. Cellulose isolated from hemp has been dissolved in N-methylmorpholine-N-oxide and subsequently spun into fibers, with mechanically treated samples producing heterogeneous spinning solutions and bleached cellulose producing homogeneous solutions [7]. These findings illustrate the importance of treatment history in determining the suitability of recovered cellulose for subsequent processing. They also provide context for investigating cellulose from hemp hurds, the woody fraction considered in the present study, and examining its characteristics after chemical treatment and sonication.

Efficient nanocellulose production requires careful control of processing conditions. Chemical pretreatment facilitates the removal of non-cellulosic components and increases cellulose accessibility, whereas subsequent mechanical treatments such as sonication or homogenization promote fibrillation and reduction in fiber dimensions [8,9]. Most nanocellulose extraction routes are described as sequential chemo-mechanical processes; however, achieving extensive fibrillation may require repeated mechanical treatment. For example, Samsalee et al. demonstrated that increasing high-pressure homogenization from six to 12 cycles affected particle size, crystallinity, and other properties of rice-husk-derived nanocellulose, while Sonyeam et al. similarly showed that the number of homogenization passes influenced fibrillation and crystallinity of nanocellulose produced from palm empty fruit bunch [9,10]. Nevertheless, these studies primarily considered repeated passes within a specific mechanical fibrillation step. In contrast, repetition of the overall extraction sequence has rarely been treated as an independent process variable in optimization of nanocellulose production from agricultural residues. In the present study, this repetition is defined as the number of extraction cycles and is introduced as an RSM factor. This variable is also relevant from an energy-efficiency perspective, because insufficient repetition may lead to incomplete fibrillation, whereas excessive mechanical processing increases energy input and may even adversely affect structural order or crystallinity [9,11]. In addition, the present extraction route employs citric acid rather than the more conventional sulfuric-acid hydrolysis, offering potential environmental and process advantages. Sulfuric-acid-based nanocellulose production is effective but can involve severe equipment corrosion, substantial water consumption for washing and neutralization, and acidic effluent generation [12,13]. By comparison, citric acid is a biodegradable organic acid that has been proposed as a more sustainable alternative for nanocellulose production and can be integrated into biorefinery concepts [14]. Importantly, citric-acid-based systems also offer the possibility of acid recovery and reuse; recyclable citric-acid-containing hydrolysis systems have been demonstrated experimentally, and citric acid can also be recovered by recrystallization from process effluents [15,16]. Thus, combining recoverable organic-acid treatment with optimization of cycle number provides an opportunity to improve both fibrillation efficiency and process sustainability.

Response surface methodology (RSM) offers an appropriate approach for simultaneously evaluating such interacting process variables and identifying operating conditions that balance competing material responses. RSM has been applied to optimize nanocellulose production from different lignocellulosic feedstocks by examining variables such as treatment severity, chemical concentration, reaction time, and mechanical processing conditions [17,18,19,20]. RSM has also been used to optimize cellulose extraction from hemp bast fibers using a choline chloride–glycerol deep eutectic solvent [21]. Nevertheless, comparatively little attention has been given to extraction cycle number as an explicit RSM factor or to how source-dependent nanocellulose characteristics subsequently influence functional modification.

Although nanocellulose provides a versatile nanoscale scaffold, its intrinsic electronic conductivity is low. Functional electrochemical applications therefore generally require incorporation of conductive or electroactive components. The hydroxyl-rich surface of nanocellulose enables interaction with inorganic and metallic species through adsorption, coordination, ionic interactions, and surface modification [22,23,24]. Copper is particularly attractive because of its electrical conductivity, availability, and ability to interact with oxygen-containing groups on cellulose-based substrates. Copper-containing cellulose and nanocellulose systems have consequently been investigated for functional and conductive applications and characterized using techniques including X-ray photoelectron spectroscopy and thermal analysis [25,26]. However, the combined effects of nanocellulose source and Cu loading on the electrochemical properties of such materials remain insufficiently understood.

Copper was selected as a conductive metallic component to functionalize nanocellulose, motivated by previous demonstrations of conductive Cu/cellulose electrodes [27].

This study examines the hypothesis that the effects of extraction cycle number and sonication duration on fibril size and FTIR-derived total crystallinity index differ between hemp-hurd and corn-cob nanocellulose. It further examines whether copper incorporation lowers low-frequency impedance and increases the cyclic potential–current response relative to bare nanocellulose, with the magnitude of these changes depending on biomass source and nominal copper loading.

The objective of this study is to determine how extraction cycle number and sonication duration influence the fibril size and FTIR-derived total crystallinity index of nanocellulose obtained from hemp hurds and corn cob, and to identify suitable processing conditions for each source. A further objective is to evaluate how biomass source and nominal copper loading affect the impedance and cyclic potential–current response of the selected nanocellulose formulations, thereby assessing their potential relevance to electrochemical electrode materials.

## 2. Materials and Methods

### 2.1. Materials

Hemp hurds, the woody inner fraction of the hemp stalk, and corn cobs were used as lignocellulosic feedstocks for nanocellulose production. Hemp hurds were supplied by Hempium Life (Merzifon, Türkiye), whereas corn cobs were obtained from local producers in Rize, Türkiye. Both feedstocks were dried, ground, and sieved to obtain 70-mesh powders before chemical treatment. These powders constituted the untreated biomass references used for comparison with the extracted nanocellulose.

Citric acid monohydrate (C_6_H_8_O_7_·H_2_O; Isolab, Eschau, Germany), sodium hydroxide (NaOH; Supelco, Bellefonte, PA, USA), sodium sulfide (Na_2_S), polyvinylpyrrolidone (PVP; Sigma-Aldrich, St. Louis, MO, USA), L-ascorbic acid (≥99%; Fisher Chemical, Fair Lawn, NJ, USA), and copper(II) nitrate trihydrate (Cu(NO_3_)_2_·3H_2_O; Tekkim, Bursa, Turkey) were used as received. Distilled water was used throughout the extraction, washing, and Cu-modification procedures.

### 2.2. Experimental Design and Optimization of Nanocellulose Extraction

Response surface methodology (RSM) was used to investigate the effects of extraction cycle number and probe-sonication time on nanocellulose production from hemp hurds and corn cobs. FTIR-derived total crystallinity index (TCI) and SEM-derived fibril size were selected as response variables.

In this study, an extraction cycle was defined as repetition of the chemical–mechanical treatment sequence used for nanocellulose production. Cycle number was therefore considered as an independent processing variable rather than treating the extraction route as a single-pass procedure. Separate RSM models were established for hemp-hurd- and corn-cob-derived nanocellulose, and numerical optimization was performed to identify conditions providing high TCI and reduced fibril dimensions while minimizing unnecessary processing.

### 2.3. Nanocellulose Extraction

#### 2.3.1. Alkaline Delignification

For each biomass source, 2 g of dried and sieved material was treated with 60 mL of an alkaline sulfide solution containing 23 wt% NaOH, 25 wt% Na_2_S, and 52 wt% distilled water. The mixtures were transferred into sealed high-pressure reactors and treated at 165 °C for 3 h.

After treatment, the solid fraction was recovered by centrifugation and washed repeatedly with distilled water to remove residual chemicals and soluble degradation products. Ethanol was included in the final washing step.

#### 2.3.2. Citric Acid Treatment

Following alkaline delignification, the recovered biomass was treated with 85 wt% citric acid. The samples were transferred into sealed reactors and maintained at 110 °C for 7 h. After treatment, the samples were centrifuged and washed with distilled water until excess citric acid and soluble reaction products were removed.

The use of citric acid provides an alternative to conventional strong mineral-acid hydrolysis and offers potential advantages in terms of lower corrosiveness and the possibility of acid recovery and reuse.

#### 2.3.3. Probe Ultrasonication

Following citric acid treatment, the cellulose-rich material was dispersed in distilled water and subjected to probe ultrasonication using an UP200Ht ultrasonic homogenizer. Sonication time was varied according to the RSM experimental design.

The sample vessel was maintained in an ice-water bath during sonication to minimize temperature increases caused by acoustic cavitation. After sonication, the suspensions were centrifuged and washed with distilled water, and the recovered nanocellulose was collected for characterization and Cu modification.

The selected nanocellulose products were designated 3-120H and 3-75C, corresponding to three extraction cycles with 120 min sonication for hemp hurds and three extraction cycles with 75 min sonication for corn cob, respectively. Their FTIR spectra were compared with those of the corresponding untreated feedstocks, and their fibrillar morphology was examined by SEM. In the subsequent Cu-modification and electrochemical sections, “bare nanocellulose” refers to these extracted products before copper incorporation.

### 2.4. Copper Modification of Nanocellulose

The optimized nanocellulose obtained from each biomass source was modified with Cu using an in situ chemical reduction method adapted from Pinto et al. [28]. Copper(II) nitrate trihydrate was used as the Cu precursor, L-ascorbic acid as the reducing agent, and PVP as the stabilizing agent.

Four nominal Cu contents, 15, 25, 50, and 75 wt%, were prepared for both hemp-hurd- and corn-cob-derived nanocellulose. The required amount of Cu(NO_3_)_2_·3H_2_O was calculated stoichiometrically from its metallic Cu content, assuming 100% reduction and incorporation efficiency. These values were considered the theoretical Cu contents of the prepared samples.

For Cu incorporation, the optimized nanocellulose was dispersed in distilled water and introduced into the reaction medium containing PVP and the corresponding amount of Cu precursor. The pH was adjusted to 6–7 using NaOH, and the reaction medium was purged with nitrogen to minimize oxidation. L-ascorbic acid was then added gradually under continuous stirring.

The reduction reaction was carried out at 70 °C under vigorous stirring. During the reaction, Cu^2+^ ions were reduced by ascorbic acid, leading to the formation of Cu-containing domains associated with the nanocellulose structure. The modified nanocellulose was subsequently recovered by centrifugation, washed to remove residual soluble species, and dried prior to characterization.

Samples were designated according to biomass source and nominal Cu loading as bare nanocellulose and 15, 25, 50, and 75 wt% Cu-modified nanocellulose.

### 2.5. Structural and Morphological Characterization

Fourier-transform infrared (FTIR) spectra were recorded using a Nicolet Apex spectrometer in attenuated total reflectance (ATR) mode to evaluate the chemical characteristics and relative structural order of the extracted nanocellulose. The total crystallinity index (TCI) was calculated according to the infrared ratio proposed by Nelson and O’Connor, using the absorbance ratio of the cellulose bands at approximately 1372 and 2900 cm^−1^ [29]:

TCI = A_1372_/A_2900_

where A_1372_ and A_2900_ represent the absorbance intensities at approximately 1372 and 2900 cm^−1^, respectively. The TCI was used as a relative, semiquantitative indicator of cellulose structural order rather than an absolute crystallinity percentage, in accordance with recent assessments of cellulose crystallinity determination methods [30].

The lateral order index (LOI) and hydrogen bonding index (HBI) were calculated as A_1430_/A_897_ and A_3340_/A_1330_, respectively, where A denotes baseline-corrected absorbance at the specified wavenumber in cm^−1^ [31]. The spectra were processed using the same settings described for TCI determination: Savitzky–Golay smoothing with an 11-point window and a third-order polynomial, and lower-envelope rubber-band baseline correction over 800–3800 cm^−1^. Absorbance heights at the specified wavenumbers were obtained by linear interpolation. The complete numerical results and corresponding baseline and band-height plots are provided in the Supplementary Materials.

Scanning electron microscopy (SEM, ZEISS EVO40) was used to examine the morphology and fibrillation of the extracted materials. Fibril dimensions were determined from SEM micrographs and used together with FTIR-derived TCI as response variables in the RSM optimization.

Following electrochemical evaluation, the 50 wt% Cu-modified formulations from each biomass source, H-NC-Cu 50 wt% and C-NC-Cu 50 wt%, were selected for further structural characterization by X-ray diffraction (XRD). XRD was used to identify the crystalline Cu-containing phase and to evaluate the remaining cellulose-related diffraction features in the Cu-modified nanocellulose.

### 2.6. Thermogravimetric Analysis and Evaluation of Cu-Containing Residue

Thermogravimetric analysis (TGA) was performed using a NETZSCH TG 309 Libra analyzer to evaluate the thermal decomposition behavior and final residual mass of the nanocellulose samples. Bare nanocellulose and samples containing nominal Cu loadings of 15, 25, 50, and 75 wt% were subjected to controlled thermal decomposition. Approximately 5–10 mg of dried sample was used for each measurement. The samples were heated from 30 to 105 °C at a heating rate of 10 °C min^−1^ and held at 105 °C for 30 min to remove residual moisture. They were subsequently heated from 105 to 600 °C at 10 °C min^−1^ and maintained at 600 °C for 60 min, followed by heating to 800 °C at the same rate and a final isothermal hold at 800 °C for 30 min. Nitrogen was supplied as a protective gas during the measurement.

The residual mass at the end of the final isothermal hold at 800 °C was recorded as the total nonvolatile residue. This residue may include Cu-containing products, carbonaceous material, and other nonvolatile constituents. For comparison among formulations, a conditional CuO-equivalent Cu content was calculated by multiplying the residual mass fraction by the molar-mass ratio M(Cu)/M(CuO), assuming that the entire residue consisted of CuO. This conversion does not establish the residue composition or independently quantify the elemental Cu content.

### 2.7. Electrochemical Characterization

Electrochemical measurements were performed using a Metrohm Autolab potentiostat coupled to Metrohm DropSens screen-printed carbon electrodes. The screen-printed electrodes (model 110) incorporated a carbon working electrode with a diameter of 4 mm, a carbon counter electrode, and a silver reference electrode. Bare nanocellulose and the four Cu-modified compositions containing nominal Cu contents of 15, 25, 50, and 75 wt% were evaluated separately for both hemp-hurd- and corn-cob-derived nanocellulose.

Three independently prepared samples were analyzed for each Cu-modified composition from each biomass source to evaluate reproducibility (*n* = 3). One bare nanocellulose control from each source was measured as a descriptive reference (*n* = 1). Equivalent amounts of sample were deposited and compacted onto the active area of the screen-printed electrodes under comparable conditions.

Electrochemical impedance spectroscopy (EIS) was performed in 0.1 M KHCO_3_ electrolyte over a frequency range of 0.1 Hz–100 kHz, using an AC perturbation amplitude of 10 mV and an applied DC potential of 0 V. Identical measurement conditions were used for all samples. Impedance magnitude, phase response, and Nyquist plots were analyzed to evaluate changes in the electrochemical response following Cu incorporation.

Cyclic potential–current measurements were additionally performed by repeatedly sweeping the applied potential between the selected negative and positive potential limits. The resulting current–potential curves were used to evaluate the electrochemical response and the charge passed during each cycle. Measurements obtained after stabilization of the cyclic response were used for comparison among compositions.

The electrochemical behavior was compared both as a function of Cu loading within each biomass source and between hemp-hurd- and corn-cob-derived nanocellulose, enabling evaluation of source-dependent differences in the electrochemical response to Cu modification.

The internal consistency of the EIS spectra was assessed using linear Kramers–Kronig (Lin-KK) measurement-model fitting [32,33]. Each of the 26 individual spectra was analyzed separately using all 61 measured frequency points between 0.1 Hz and 100 kHz. Modulus-weighted complex regression was supplemented by real-component regression and model-order sensitivity checks. The fitting procedure, residual definitions, and individual numerical results are provided in the Supplementary Materials.

## 3. Results

### 3.1. RSM-Guided Optimization of Nanocellulose Extraction

**Figure 1:**
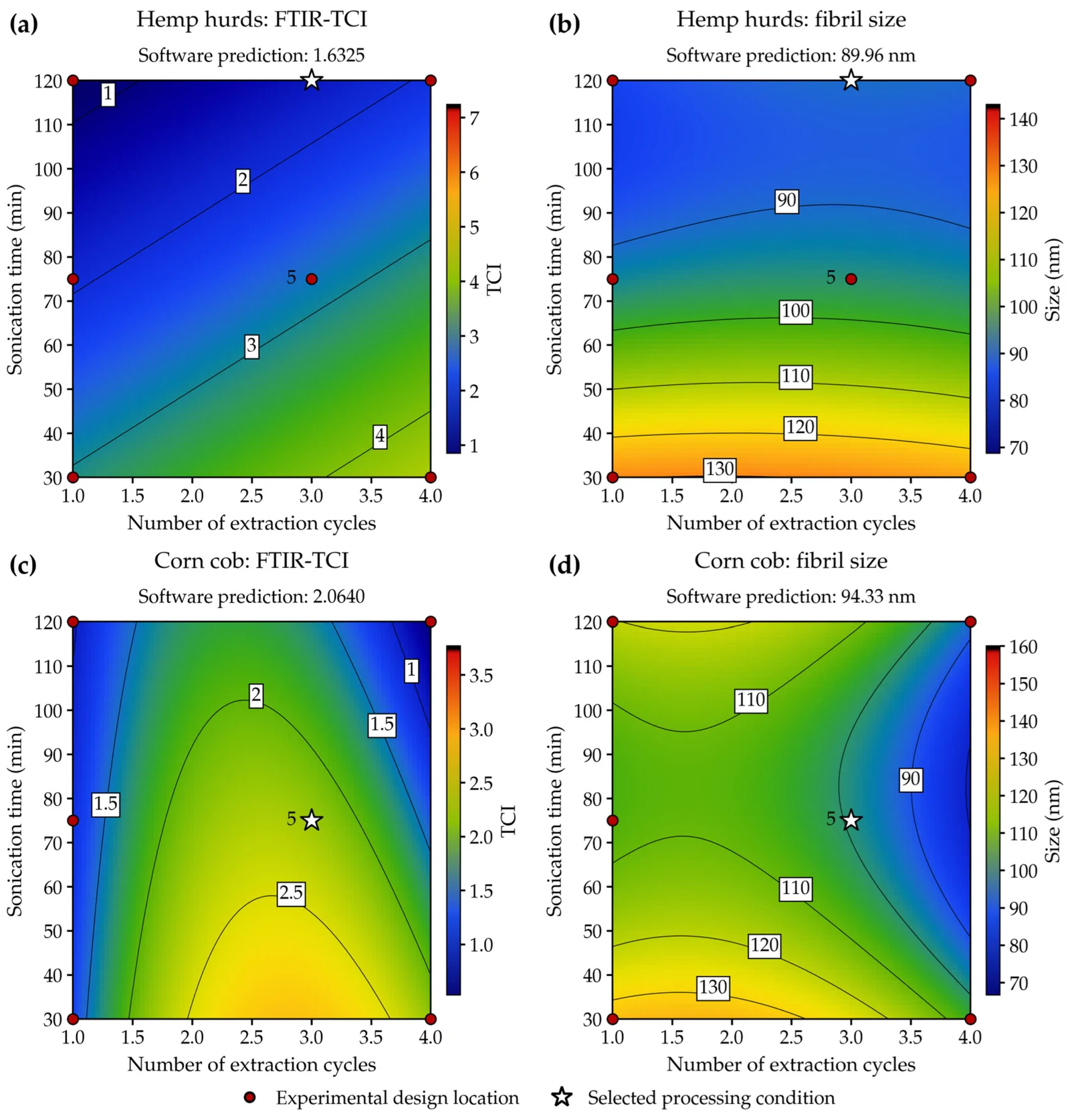
Model-predicted contour maps for FTIR-derived total crystallinity index (TCI) and apparent fibril size as functions of extraction cycle number and sonication time: (**a**) hemp-hurd TCI, (**b**) hemp-hurd fibril size, (**c**) corn-cob TCI, and (**d**) corn-cob fibril size. Red circles indicate experimental design locations within the plotted factor domain; the label 5 denotes five overlapping central runs. White stars indicate the processing conditions selected for subsequent Cu modification: three cycles and 120 min for hemp hurds (3-120H), and three cycles and 75 min for corn cob (3-75C).

Nanocellulose extraction from hemp hurds and corn cob was evaluated as a two-factor process in which extraction cycle number and probe-sonication time were varied, with FTIR-derived total crystallinity index (TCI) and SEM-derived fibril size used as responses. The two feedstocks exhibited different response landscapes. TCI was treated as a relative band-intensity-based indicator of cellulose structural order rather than as an absolute crystallinity percentage [30]. The model-predicted response surfaces are presented in Figure 1. The complete design matrices, individual response values, model outputs, numerical optimization results, and supporting SEM and FTIR analyses are provided in the Supplementary Materials.

#### 3.1.1. Hemp-Hurd Nanocellulose

The hemp-hurd experiments yielded FTIR-derived total crystallinity index (TCI) values ranging from 0.851 to 7.239 and SEM-derived apparent fibril widths ranging from 68.7 to 143 nm. The two responses varied differently with extraction cycle number and sonication time, emphasizing the importance of considering them together when selecting the processing conditions. At the central condition of three extraction cycles and 75 min sonication, the five experimental runs yielded a mean TCI of 3.636 ± 0.071 and a mean apparent fibril width of 97.74 ± 1.96 nm (mean ± sample standard deviation, *n* = 5). The small dispersion among these runs indicated consistent measured responses at this condition.

At one extraction cycle, TCI remained within a narrow range of 1.061–1.172 across sonication times of 30–120 min. However, the corresponding fibril widths varied appreciably: increasing sonication from 30 to 75 min reduced the measured width from 143 to 76.7 nm, whereas extending sonication to 120 min yielded a width of 86.7 nm. Thus, longer sonication did not produce a continuous reduction in fibril width. Similarly, at 75 min sonication, increasing the extraction cycle number from one to three and five yielded TCI values of 1.061, 3.636 on average, and 3.315, respectively, while the corresponding widths were 76.7, 97.74 on average, and 97 nm. These observations showed that changes in the FTIR-derived index were not accompanied by proportional changes in fibril width.

The highest TCI in the experimental table, 7.239, was obtained after three extraction cycles and 11 min sonication, with a corresponding fibril width of 140 nm. At three cycles and 140 min sonication, TCI was 0.851 and the measured width was 110 nm. Conversely, the smallest width in the experimental table, 68.7 nm, was obtained after four cycles and 120 min sonication, but its TCI was 1.098. Therefore, the conditions giving the highest TCI and the smallest fibril width did not coincide.

Following comparison of alternative model forms, linear and quadratic models were used for TCI and fibril width, respectively. Numerical optimization selected three extraction cycles and 120 min sonication, with predicted responses of 1.6325 for TCI and approximately 90.0 nm for fibril width, and a composite desirability of 0.894. Experimental characterization of the corresponding 3-120H sample yielded a TCI of 5.735 and a mean apparent fibril width of 83 nm. The measured width was approximately 6.7 nm, or 7.4%, below the predicted value, whereas the experimental TCI exceeded the model prediction.

The 3-120H condition combined a relatively high measured TCI with a small measured fibril width. Compared with the four-cycle, 120 min condition, it yielded a higher TCI while requiring one fewer extraction cycle, although its fibril width was larger. Compared with the three-cycle, 11 min condition, it yielded a substantially smaller width while retaining a relatively high TCI. This balance of experimentally measured responses supported the selection of 3-120H for subsequent Cu modification (Table 1).

#### 3.1.2. Corn-Cob Nanocellulose

The corn-cob experiments yielded FTIR-derived total crystallinity index (TCI) values ranging from 0.530 to 3.768 and SEM-derived apparent fibril widths ranging from 66.7 to 160 nm. The responses showed different dependencies on extraction cycle number and sonication time, requiring their combined evaluation when selecting the processing conditions. At the central condition of three extraction cycles and 75 min sonication, the five experimental runs yielded a mean TCI of 2.242 ± 0.002 and a mean apparent fibril width of 99.6 ± 2.1 nm (mean ± sample standard deviation, *n* = 5). The small dispersion among these runs indicated consistent measured responses at this condition.

At one extraction cycle, increasing sonication time from 30 to 75 and 120 min yielded TCI values of 1.071, 1.106, and 1.156, respectively. The corresponding fibril widths decreased from 130 to 113.3 nm between 30 and 75 min and remained at 113.3 nm after 120 min. Thus, extending sonication beyond 75 min did not further reduce the measured width at this cycle number. At 75 min sonication, increasing the extraction cycle number from one to three increased TCI from 1.106 to an average of 2.242, while the average width decreased from 113.3 to 99.6 nm. Increasing the treatment to five cycles yielded a smaller width of 80 nm but a lower TCI of 0.530. Therefore, additional extraction cycles did not consistently improve both responses.

At three extraction cycles, the shortest sonication time, 11 min, yielded the highest measured TCI, 3.768, together with the largest fibril width, 160 nm. At 75 min, the average width was smaller, whereas extending sonication to 140 min yielded a width of 146 nm and a TCI of 1.123. The measured width therefore varied non-monotonically with sonication time. At four extraction cycles, increasing sonication from 30 to 120 min reduced the width from 83.3 to 66.7 nm, but TCI also decreased from 1.292 to 0.540. These results showed that the smallest measured fibril width did not coincide with the highest TCI.

Quadratic response models were used for TCI and fibril width. Numerical optimization identified three extraction cycles and 75 min sonication as the predicted optimum, with a predicted fibril width of 94.3 nm, a predicted TCI of approximately 2.06, and a composite desirability of 0.713. Experimental characterization of the selected 3-75C sample yielded a mean apparent fibril width of 99.6 ± 2.1 nm (*n* = 5) and a TCI of 2.242 ± 0.002. These measured responses differed from the predictions by approximately 5.3 nm and 0.18 TCI units, respectively, showing close numerical agreement at the selected processing condition.

The 3-75C condition combined a relatively high measured TCI with a smaller fibril width than the condition yielding the maximum TCI. Although more extensive processing produced smaller widths in some experiments, these conditions also yielded lower TCI values. The measured balance between the two responses, together with the numerical optimization result, supported the selection of 3-75C for subsequent Cu modification (Table 1).

**Figure 2:**
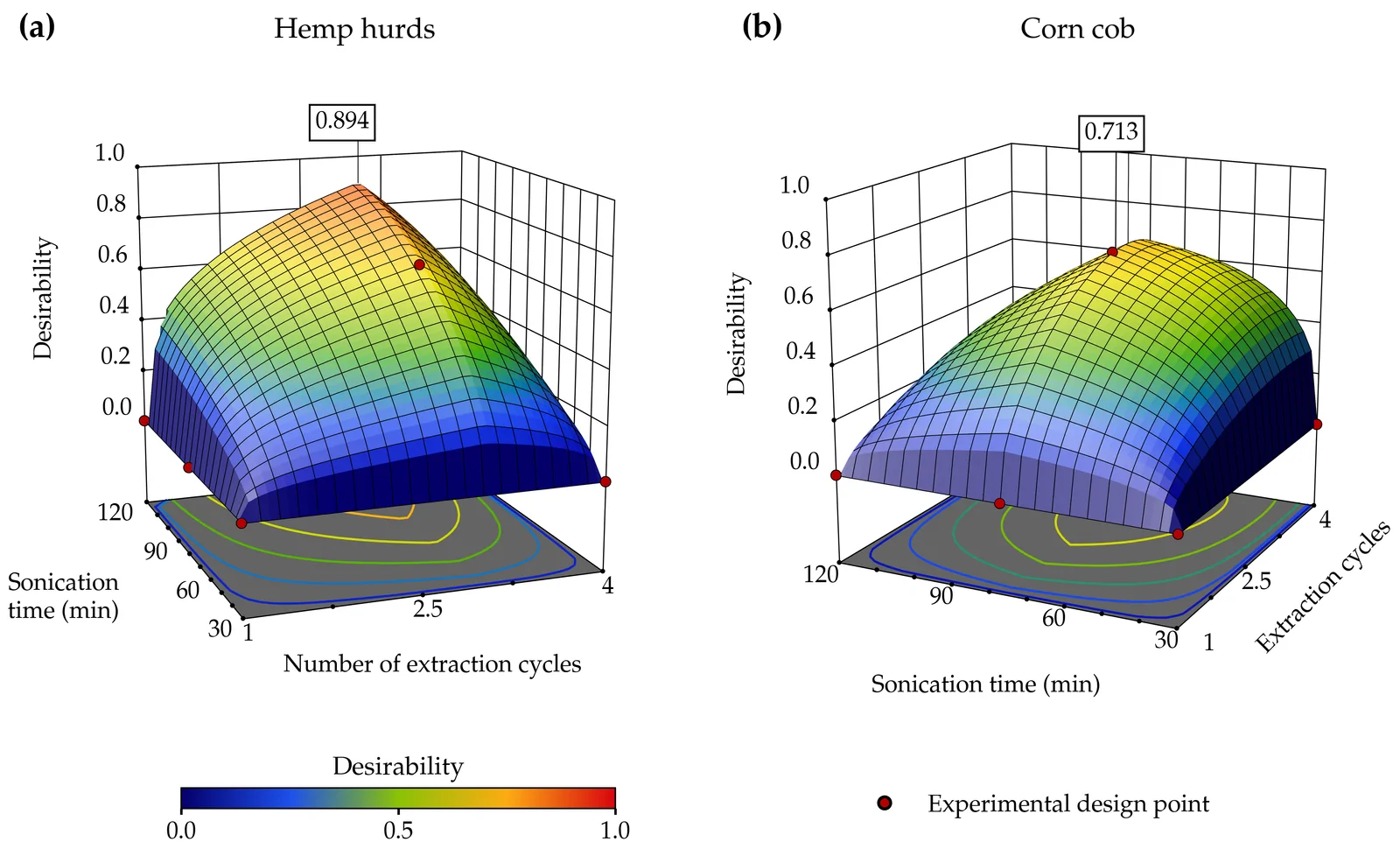
Three-dimensional desirability response surfaces for nanocellulose extraction optimization: (**a**) hemp hurds and (**b**) corn cob. The optimum regions correspond to 3-120H for hemp hurds and 3-75C for corn cob.

The three-dimensional desirability surfaces further illustrate the distinct optimization windows of the two biomass sources (Figure 2). For hemp hurds, the desirability maximum was concentrated at high sonication time and an intermediate cycle number, supporting the selection of three cycles and 120 min as the optimum condition. In contrast, corn cob exhibited a broader optimum region, with the desirability maximum centered around three cycles and 75 min.

#### 3.1.3. Model Adequacy

The relationships between extraction conditions and nanocellulose properties were described using a linear model for hemp-hurd FTIR-derived total crystallinity index (TCI) and a quadratic model for fibril size. Quadratic models were used for both corn-cob responses. The linear formulation describes the individual factor effects, whereas quadratic formulations allow the representation of curvature and interactions between extraction cycle number and sonication time. Model fit was assessed using the coefficient of determination (R^2^), which expresses the proportion of observed response variability explained by the model, and adjusted R^2^, which accounts for the number of fitted terms. The fibril-size models yielded R^2^ values of 0.7833 for hemp hurds and 0.7364 for corn cob.

Comparison with experimental measurements showed reasonable agreement in fibril size at the selected processing conditions. For hemp hurds, three extraction cycles and 120 min sonication (3-120H) gave a predicted fibril size of 89.96 nm, compared with the SEM-derived mean of 83.3 nm. For corn cob, three cycles and 75 min sonication (3-75C) gave a predicted size of 94.33 nm, compared with the experimental mean of approximately 100 nm. The corn-cob TCI prediction of 2.064 was also close to the experimental value of 2.242, whereas hemp-hurd TCI showed a larger difference, with an experimental value of 5.735 compared with the predicted value of 1.6325. The correspondence therefore varied with the response and feedstock, with closer agreement obtained for fibril size in both materials and for corn-cob TCI. These comparisons support the practical selection of the processing conditions, while indicating that the hemp-hurd TCI estimate should be interpreted alongside the experimental FTIR measurements.

#### 3.1.4. FTIR and SEM Confirmation of the Selected Nanocellulose

**Figure 3:**
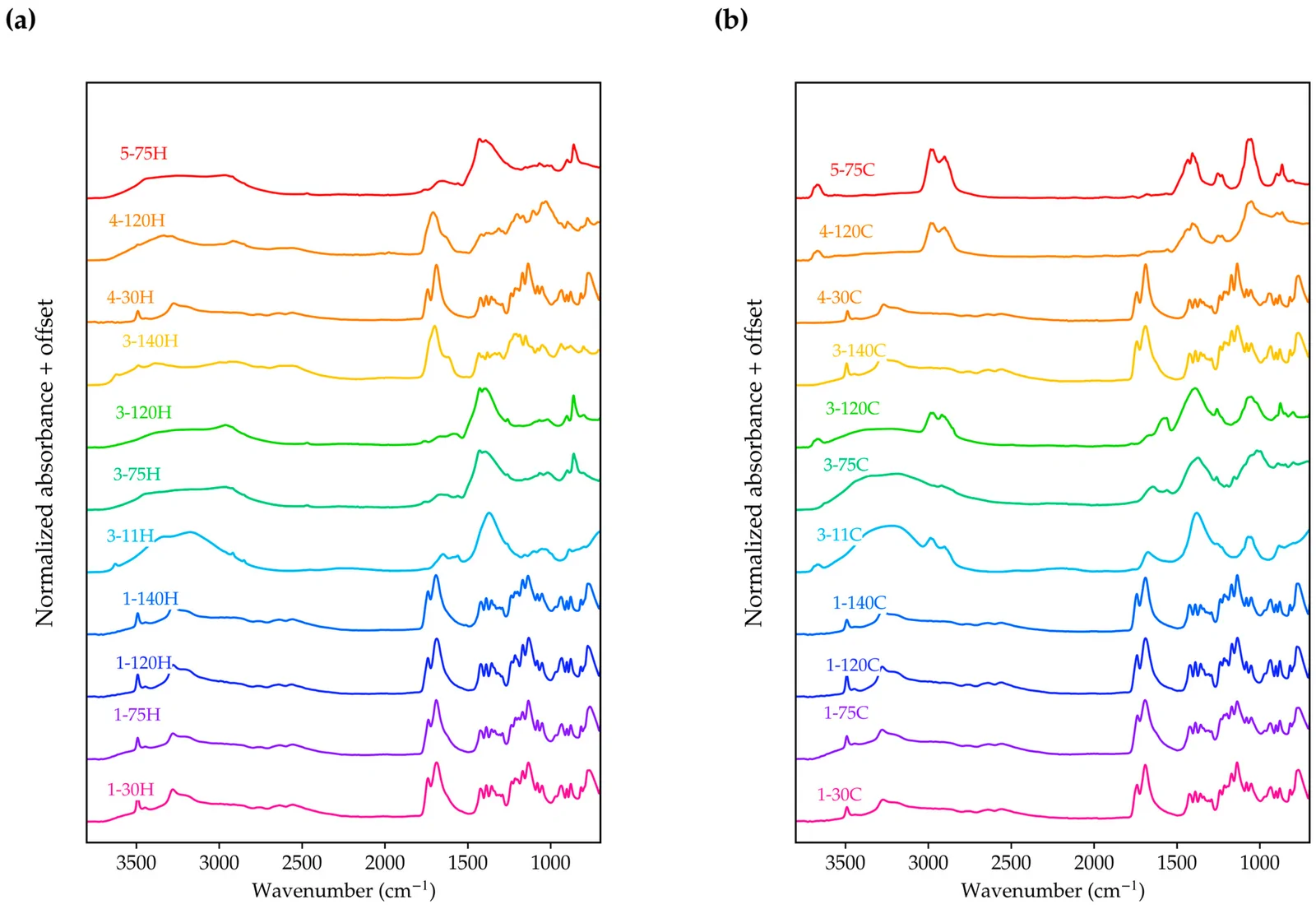
Stacked, baseline-corrected FTIR spectra of (**a**) hemp-hurd- and (**b**) corn-cob-derived nanocellulose prepared under the investigated extraction conditions. Spectra were individually normalized and vertically offset for visualization.

The stacked FTIR spectra (Figure 3) show that the main cellulose-related spectral features were retained across the extraction conditions for both biomass sources, while relative band intensities varied with processing. Complementary to the TCI analysis, the selected hemp-hurd sample, 3-120H, exhibited LOI and HBI values of 17.649 and 0.396, respectively, whereas the selected corn-cob sample, 3-75C, exhibited values of 3.211 and 1.133. These FTIR-derived indices were used for relative comparison under the stated preprocessing procedure and were not interpreted as absolute crystallinity percentages or directly equated with XRD-derived crystallinity values [30]. The complete LOI and HBI results for the 22 spectra, together with plots illustrating the baseline and band heights used for the FTIR-index calculations, are provided in the Supplementary Materials.

**Figure 4:**
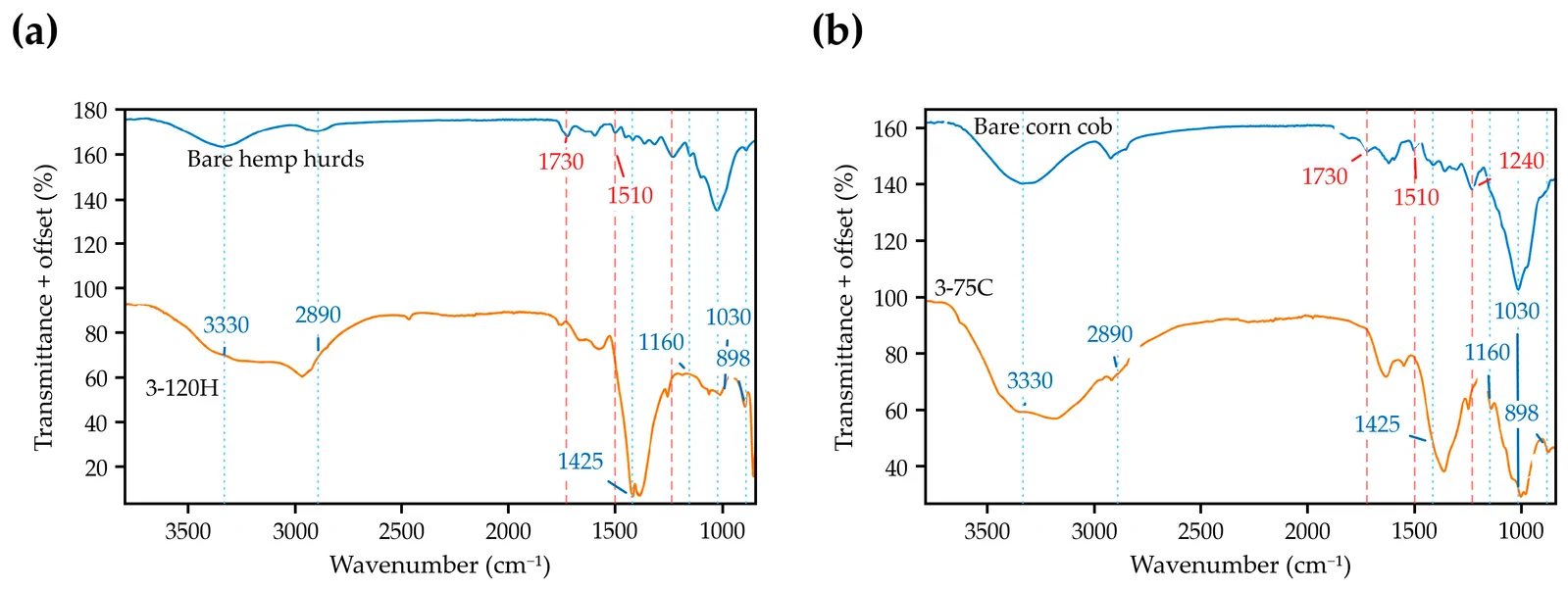
FTIR transmittance spectra of the untreated lignocellulosic feedstocks and the corresponding optimized nanocellulose samples: (**a**) hemp hurds, comparing untreated hemp hurds with the optimized 3-120H sample, and (**b**) corn cob, comparing untreated corn cob with the optimized 3-75C sample.

Comparison of untreated biomass with the selected nanocellulose samples (Figure 4) showed the broad O–H stretching band near 3330 cm^−1^, C–H stretching around 2890 cm^−1^, CH_2_ bending near 1425 cm^−1^, asymmetric C–O–C stretching around 1160 cm^−1^, C–O stretching near 1030 cm^−1^, and the band around 898 cm^−1^ associated with β-glycosidic linkages, all characteristic of cellulose-based materials [4,18,19]. Relative to the untreated feedstocks, the optimized samples showed strong attenuation of the band near 1730 cm^−1^, a marked decrease around 1510 cm^−1^, and substantial reduction in the feature near 1240 cm^−1^, which are commonly associated with hemicellulose carbonyl groups and lignin-related aromatic/C–O vibrations; their reduction is consistent with removal of non-cellulosic constituents during treatment [18,19].

**Figure 5:**
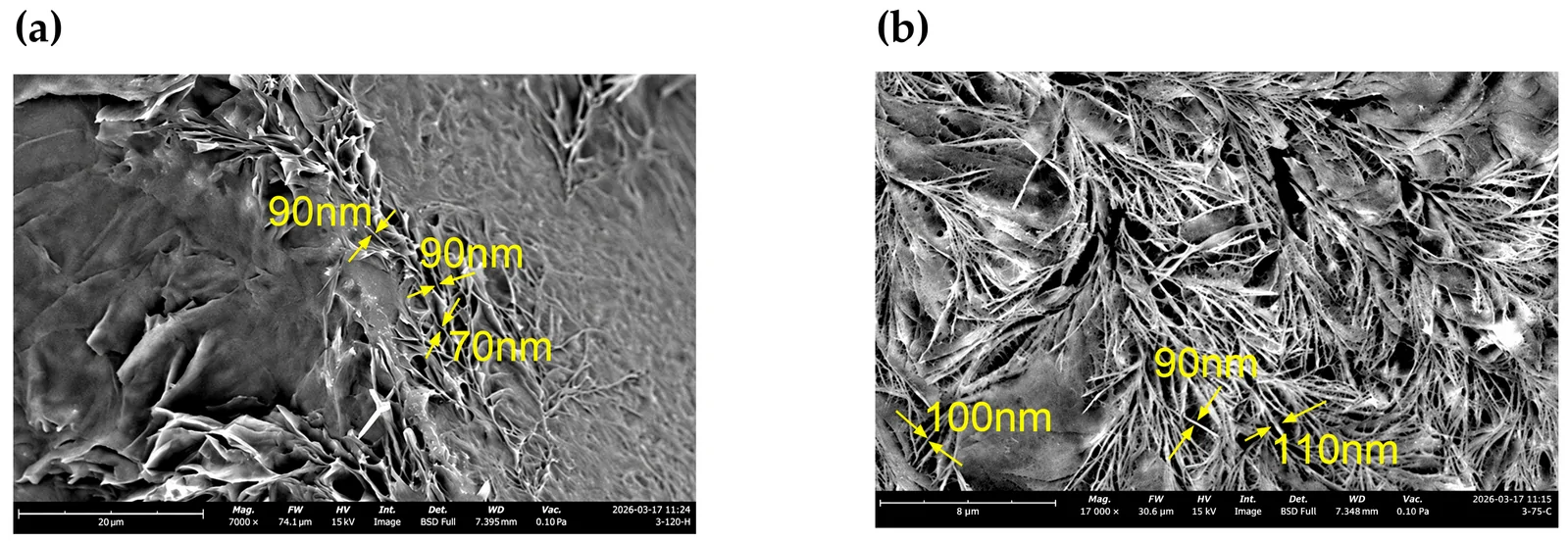
Representative SEM images of the nanocellulose samples selected for subsequent Cu modification: (**a**) hemp-hurd sample 3-120H and (**b**) corn-cob sample 3-75C. Scale bars and measurement annotations are retained from the supplied micrographs.

Representative SEM images (Figure 5) showed extensive fibrillation in both selected samples. The 3-120H field contained fine fibrillar regions with annotated apparent widths of approximately 70–90 nm, whereas 3-75C showed a dense fibrillar network with annotated features around 90–110 nm in the presented field. The complete SEM dataset for all extraction experiments is provided in the Supplementary Materials.

Together, the FTIR comparison of the untreated feedstocks and extracted products (Figure 4) and the SEM observations (Figure 5) characterize the materials before and after the extraction sequence. The selected hemp-hurd and corn-cob products exhibited measured mean fibril sizes of 83.3 nm and approximately 100 nm, respectively, with corresponding FTIR-derived TCI values of 5.735 and 2.242. These TCI values describe relative infrared band-intensity ratios and are not absolute crystallinity percentages.

### 3.2. Electrochemical Characterization of Cu-Modified Hemp-Hurd and Corn-Cob Nanocellulose

Bare nanocellulose and samples containing nominal Cu loadings of 15, 25, 50, and 75 wt% were evaluated by electrochemical impedance spectroscopy (EIS) and cyclic potential–current measurements in 0.1 M KHCO_3_ electrolyte. EIS measurements were performed over a frequency range of 0.1 Hz–100 kHz using an AC perturbation amplitude of 10 mV and an applied DC potential of 0 V. Three independently prepared specimens were analyzed for each Cu-modified composition (*n* = 3). Each corresponding bare nanocellulose control was measured once (*n* = 1) and treated as a descriptive reference, excluding it from inferential statistics. Results for the Cu-modified groups are reported as arithmetic means ± sample standard deviations. Total passed charge was calculated for each specimen and cycle as Q^+^ + |Q^−^| before calculating group statistics. Exploratory one-way ANOVA compared the four Cu loadings within each nanocellulose source, and Tukey’s HSD was used for pairwise comparisons of the positive charge component. Two-way ANOVA evaluated nanocellulose source, Cu loading, and their interaction using the individual Cu-modified specimens.

Electrochemical measurements were performed in 0.1 M potassium bicarbonate (KHCO_3_) electrolyte. EIS spectra were recorded from 100 kHz to 0.1 Hz using an AC perturbation amplitude of 10 mV at a DC potential of 0 V, with 61 frequency points per spectrum.

#### 3.2.1. Electrochemical Impedance Spectroscopy

**Figure 6:**
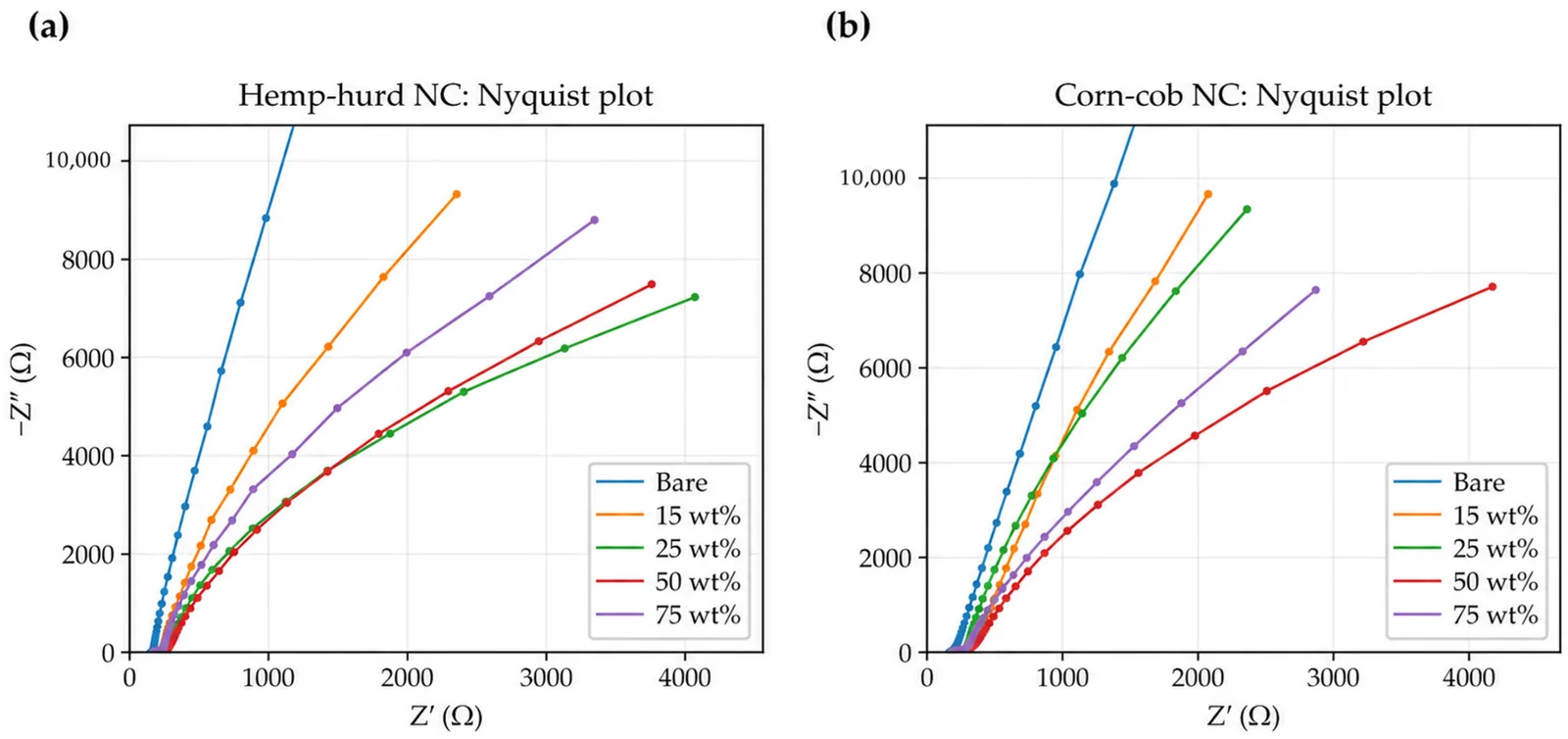
Nyquist plots of Cu-modified nanocellulose: (**a**) hemp-hurd-derived NC and (**b**) corn-cob-derived NC. The plotted ranges are restricted to preserve visibility of the Cu-modified responses; the bare controls exhibit substantially larger low-frequency impedance.

Cu incorporation produced a pronounced decrease in low-frequency impedance for both nanocellulose sources. At 0.1 Hz, bare hemp-hurd nanocellulose exhibited |Z| = 274.15 kΩ and bare corn-cob nanocellulose exhibited |Z| = 247.67 kΩ, whereas all Cu-modified formulations had mean values below 10 kΩ. The high-frequency Z′ values of the Cu-modified groups changed comparatively little and remained approximately 175–187 Ω. The Nyquist plots are shown in Figure 6.

For hemp-hurd nanocellulose, the mean |Z| values at 0.1 Hz were 9.65 ± 0.92, 8.44 ± 1.27, 8.44 ± 0.63, and 9.45 ± 0.29 kΩ for 15, 25, 50, and 75 wt% Cu, respectively. One-way ANOVA across the four Cu-modified groups gave *p* = 0.245. The 25 wt% group showed the largest variability in low-frequency impedance, and all three independently prepared specimens were retained in the analysis.

For corn-cob-derived nanocellulose, mean |Z| at 0.1 Hz decreased from 9.90 ± 0.81 kΩ at 15 wt% Cu to 9.65 ± 0.44, 8.81 ± 1.02, and 8.17 ± 0.84 kΩ at 25, 50, and 75 wt% Cu, respectively (Table 2). One-way ANOVA across the four Cu-modified groups yielded *p* = 0.103 for impedance magnitude and *p* = 0.00563 for the −phase values at 0.1 Hz.

**Figure 7:**
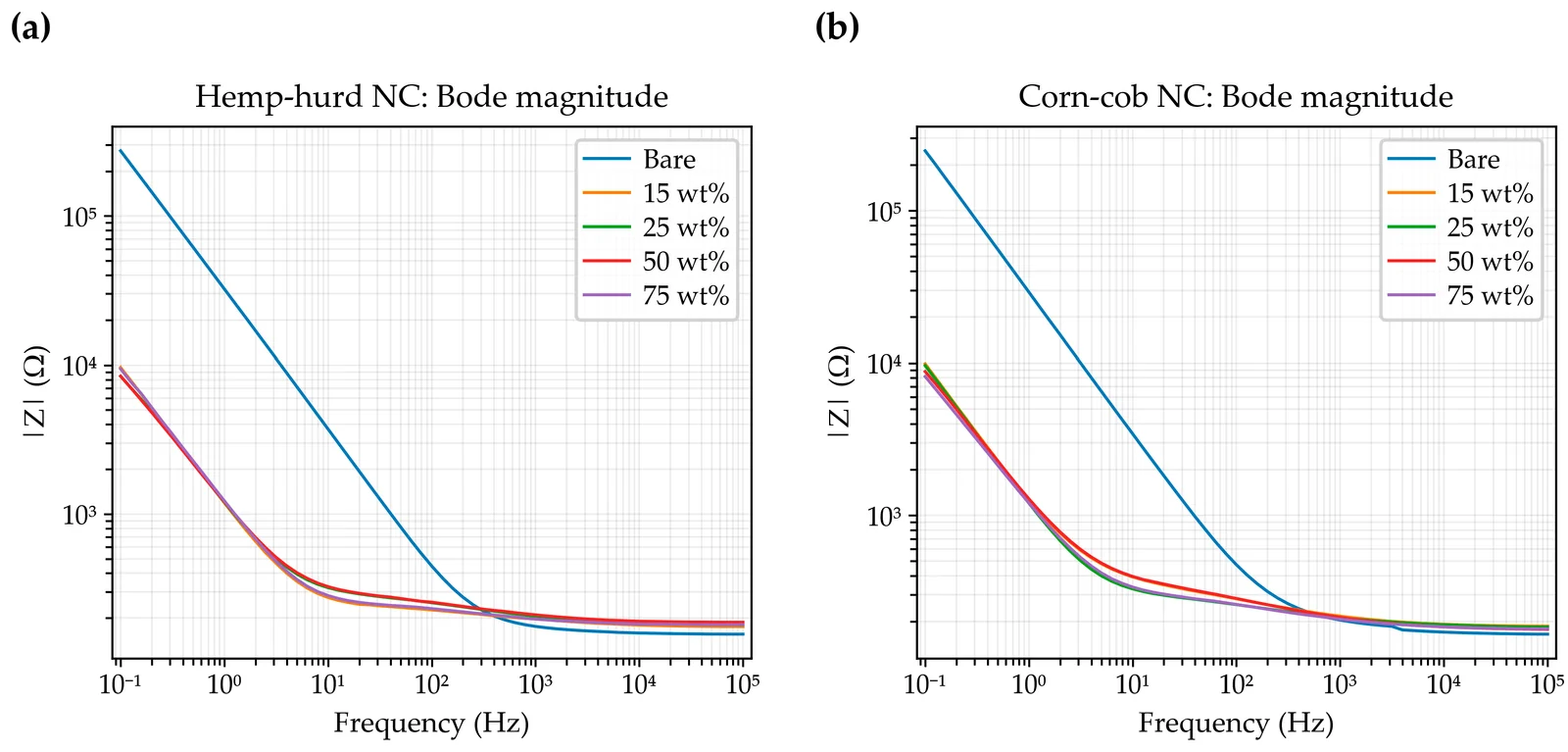
Bode impedance magnitude plots for (**a**) hemp-hurd-derived and (**b**) corn-cob-derived bare and Cu-modified nanocellulose.

**Figure 8:**
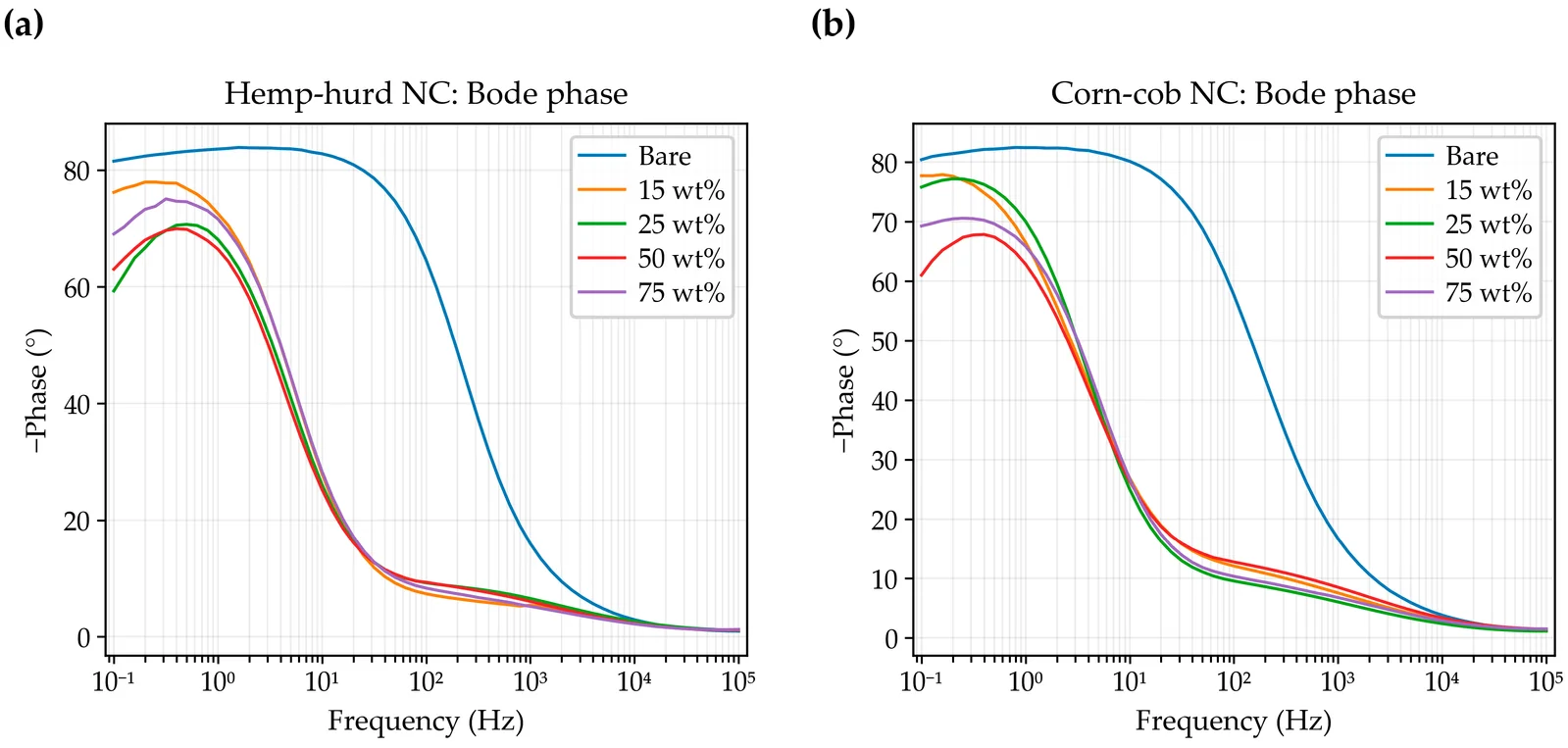
Bode phase plots for (**a**) hemp-hurd-derived and (**b**) corn-cob-derived bare and Cu-modified nanocellulose.

The Bode magnitude plots (Figure 7) showed the largest separation between bare and Cu-modified samples at low frequency. For corn cob, bare NC exhibited |Z| = 29.16 kΩ at 1 Hz, whereas the mean values of the Cu-modified groups ranged from approximately 1.20 to 1.28 kΩ. In the corn-cob series, the mean −phase value at 0.1 Hz decreased from 77.73 ± 3.35° at 15 wt% Cu to 61.03 ± 7.22° at 50 wt% Cu and then increased to 69.28 ± 2.31° at 75 wt% Cu (Figure 8).

**Figure 9:**
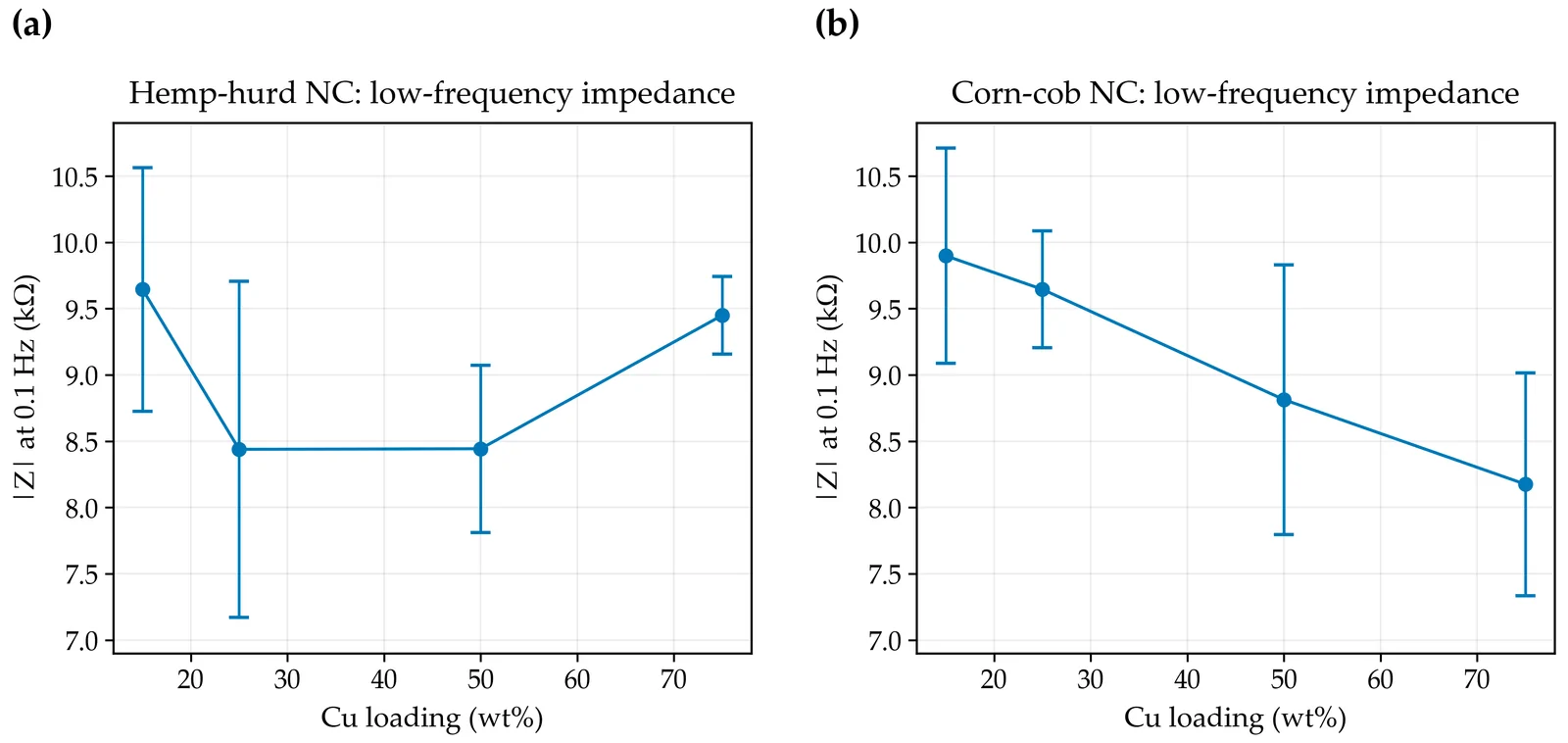
Impedance magnitude at 0.1 Hz as a function of nominal Cu loading for (**a**) hemp-hurd-derived and (**b**) corn-cob-derived Cu-modified nanocellulose. Circles represent arithmetic means and error bars represent sample standard deviations from three independently prepared specimens per group (*n* = 3).

Figure 9 summarizes the loading dependence of low-frequency impedance. The hemp-hurd series was non-monotonic, whereas the corn-cob series showed a progressive decrease from 15 to 75 wt% Cu. Equivalent-circuit fitting was investigated; however, the tested models did not establish a physically validated charge-transfer resistance under the measurement conditions. Consequently, the fitted resistances were not assigned to R_ct_.

The Lin-KK analysis yielded modulus-normalized complex root-mean-square residuals of 0.35–1.49% across the 26 spectra. The residual profiles showed frequency-dependent deviations, which were generally more pronounced near the low-frequency end of the measured range. The largest absolute component residual was 6.83% at 0.1 Hz for C-NC-Cu 50% specimen 3. The individual residual statistics, frequency-dependent residual plots, and additional regression and sensitivity checks are presented in the Supplementary Materials.

#### 3.2.2. Cyclic Potential–Current Response

**Figure 10:**
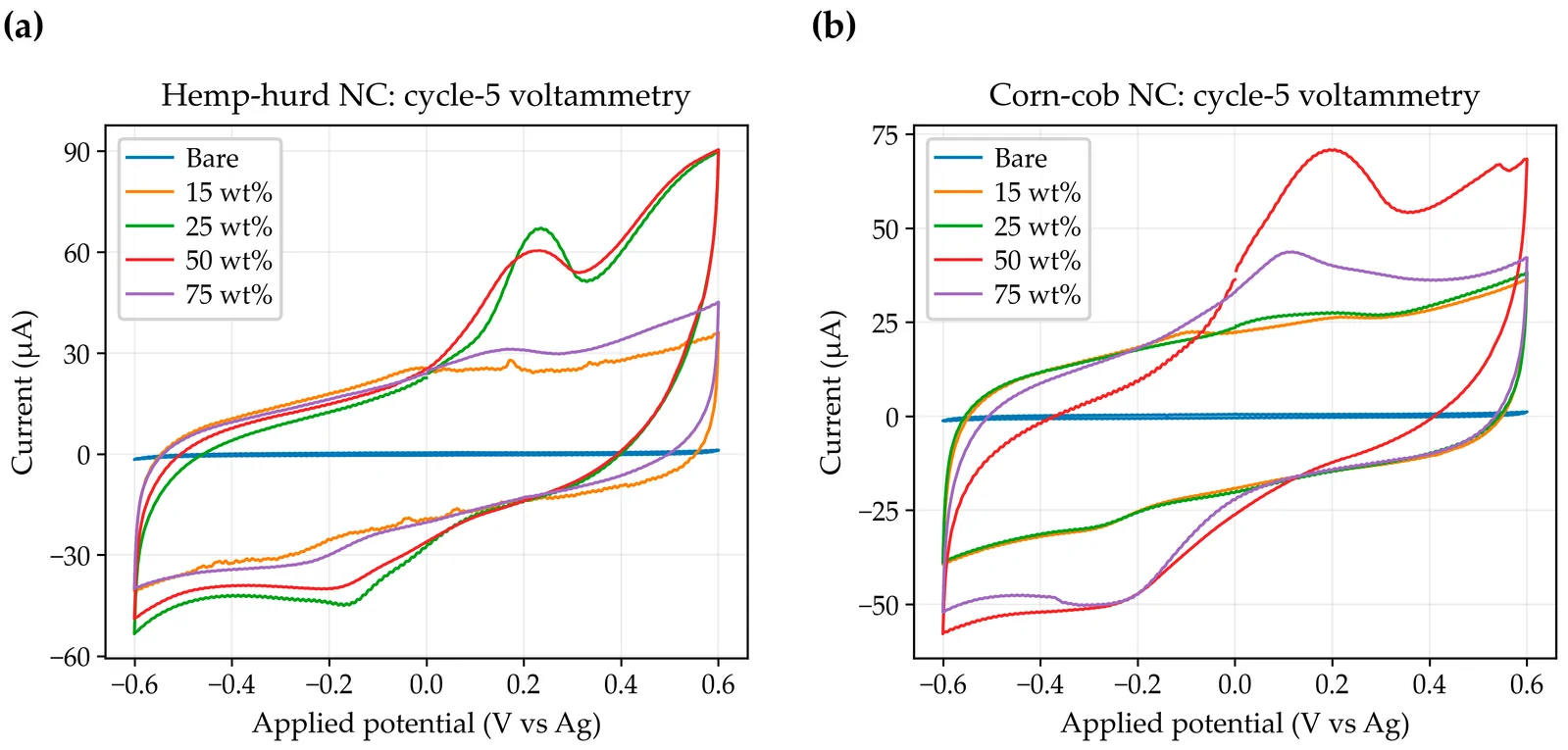
Cycle-5 potential–current curves for (**a**) hemp-hurd-derived and (**b**) corn-cob-derived bare and Cu-modified nanocellulose. Cu-modified curves represent means of three independently prepared samples.

The cyclic potential–current measurements showed a marked increase in current and passed charge after Cu incorporation. Cycle 5 was used as a common endpoint for comparison among compositions. The mean cycle-5 curves are presented in Figure 10.

**Figure 11:**
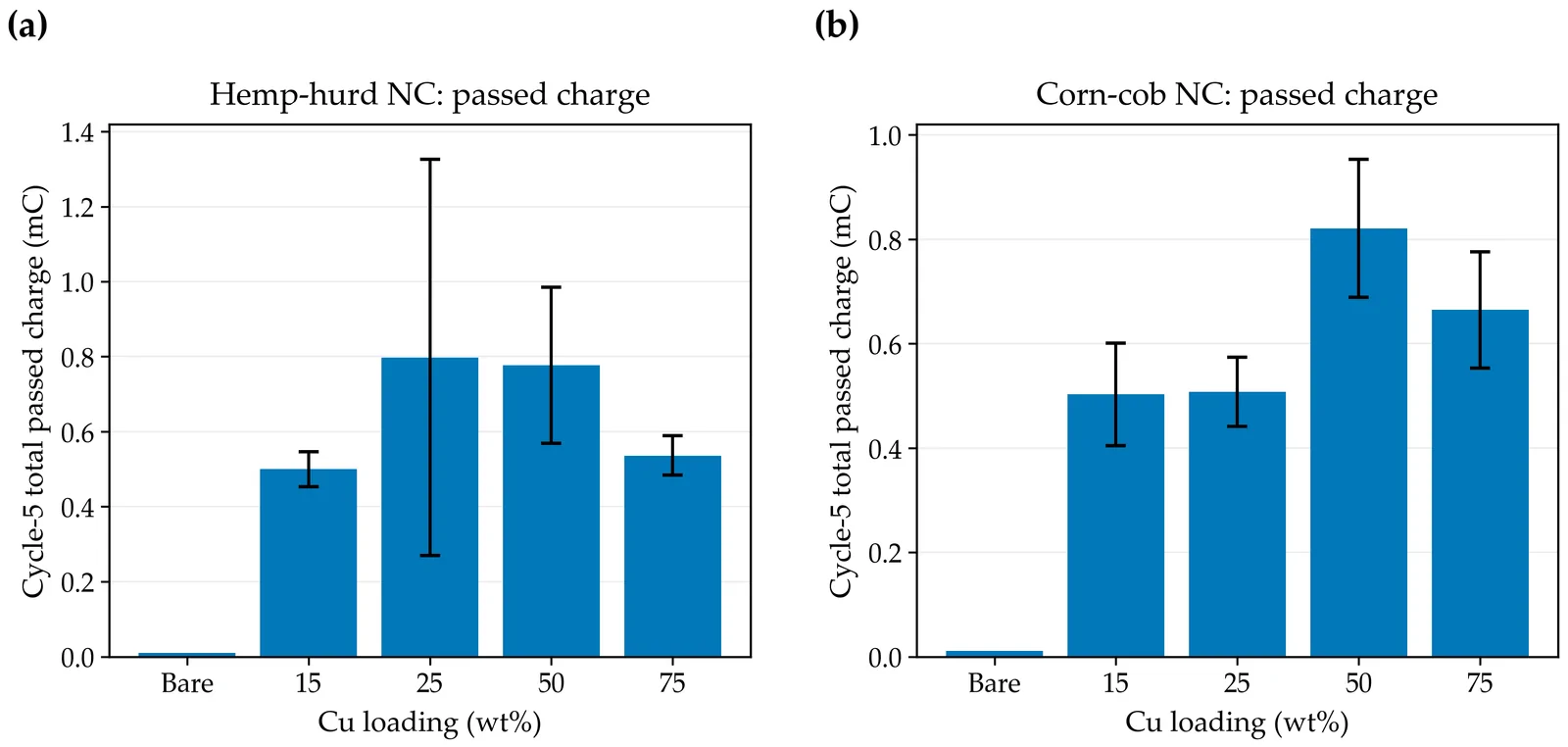
Cycle-5 total passed charge for (**a**) hemp-hurd-derived and (**b**) corn-cob-derived bare and Cu-modified nanocellulose. Error bars represent SD for the Cu-modified groups (*n* = 3). Each bare control has one recording (*n* = 1).

As shown in Figure 11, hemp-hurd-drived bare nanocellulose exhibited a cycle-5 total passed charge of 0.0104 mC. The Cu-modified groups yielded 0.500 ± 0.047 mC at 15 wt%, 0.798 ± 0.528 mC at 25 wt%, 0.777 ± 0.208 mC at 50 wt%, and 0.536 ± 0.053 mC at 75 wt% Cu. H25-2 produced 1.399 mC compared with 0.407 and 0.588 mC for H25-1 and H25-3; it changed by only −6.3% from cycle 4 to cycle 5 and was retained as an independent replicate. The one-way ANOVA across the Cu-modified hemp groups was not significant (*p* = 0.484).

For corn-cob nanocellulose, mean cycle-5 total passed charge values were 0.503 ± 0.098, 0.507 ± 0.066, 0.821 ± 0.132, and 0.665 ± 0.111 mC for 15, 25, 50, and 75 wt% Cu, respectively, compared with 0.0109 mC for bare corn-cob NC (Table 3). The highest mean response occurred at 50 wt% Cu. One-way ANOVA yielded *p* = 0.0172 for total passed charge and *p* = 0.00936 for the positive charge component, Q^+^. Tukey comparisons for Q^+^ indicated higher values at 50 wt% than at 15 and 25 wt% Cu, with adjusted *p*-values of 0.0118 and 0.0146, respectively. Hysteresis between the forward and reverse potential scans was visible in the Cu-modified samples.

**Figure 12:**
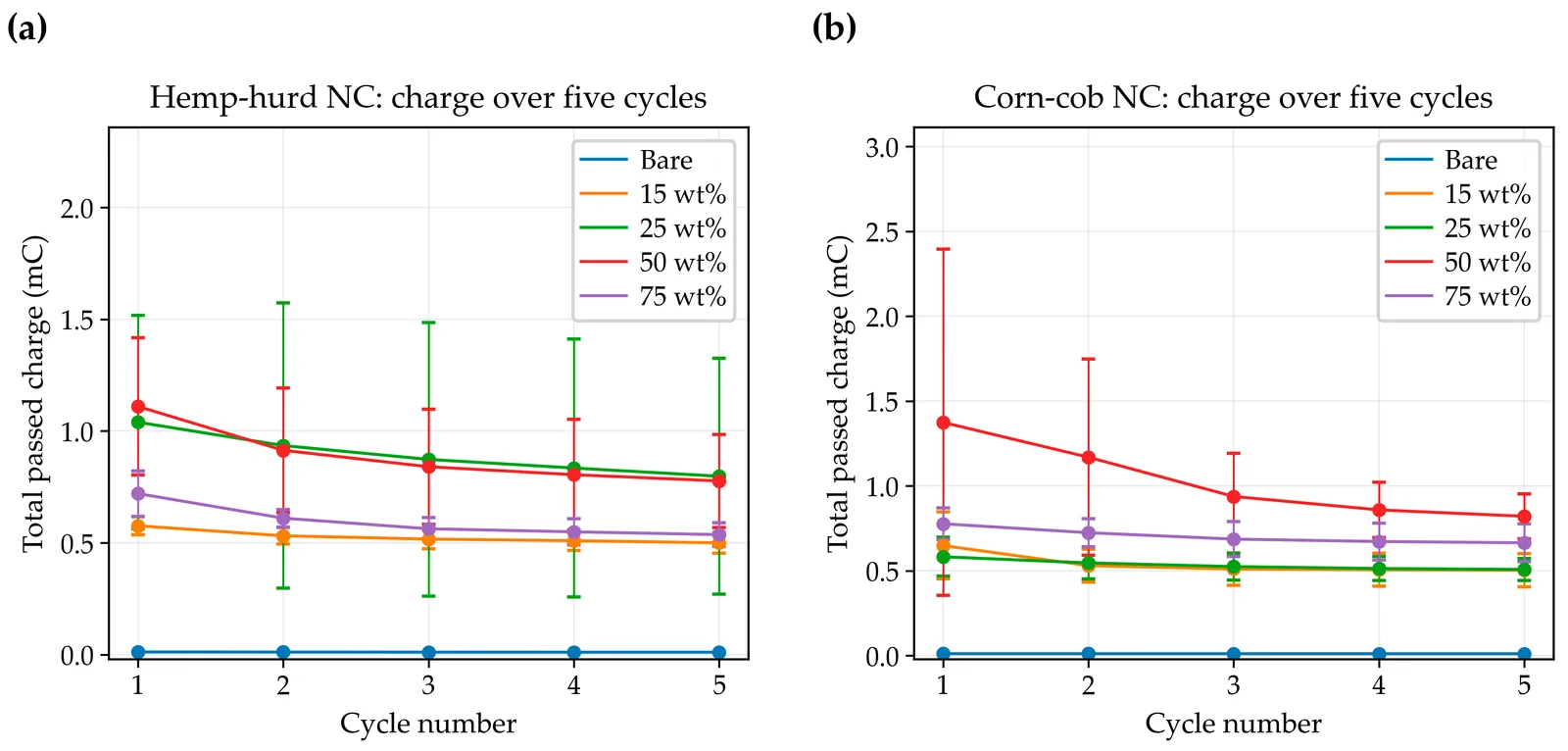
Total passed charge over five consecutive potential cycles for (**a**) hemp-hurd-derived and (**b**) corn-cob-derived bare and Cu-modified nanocellulose.

Across five consecutive cycles (Figure 12), the largest changes in mean total passed charge generally occurred during the early cycles and became smaller with further cycling. Between cycles 4 and 5, mean total passed charge decreased by approximately 1.9–4.4% for the Cu-modified hemp-hurd groups and 0.5–4.4% for the corresponding corn-cob groups. The 50 wt% corn-cob group showed a 4.4% decrease, compared with approximately 0.5–1.1% for the other Cu-modified corn-cob groups.

#### 3.2.3. Source-Dependent Electrochemical Response

**Figure 13:**
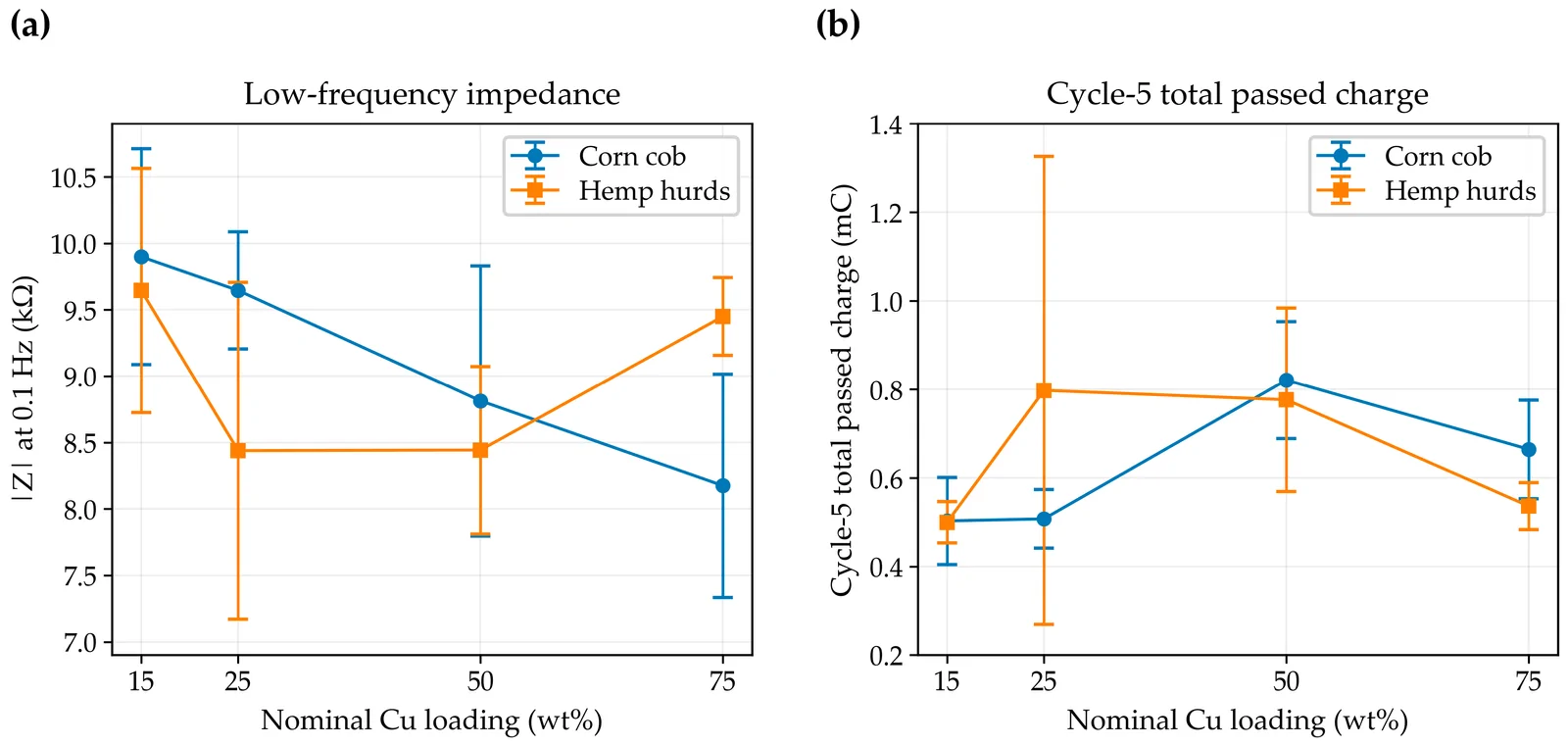
Direct comparison of hemp-hurd-derived and corn-cob-derived Cu-modified nanocellulose as a function of nominal Cu loading: (**a**) impedance magnitude at 0.1 Hz and (**b**) cycle-5 total passed charge. Values are mean ± SD for three independently prepared Cu-modified samples.

At 15 wt% Cu, the mean low-frequency impedances of the two nanocellulose sources were close. At 25 and 50 wt%, the hemp-hurd samples exhibited lower mean |Z| at 0.1 Hz, whereas at 75 wt% the corn-cob formulation exhibited the lower mean impedance (Figure 13a). Two-way ANOVA yielded *p* = 0.688 for nanocellulose source, *p* = 0.130 for Cu loading, and *p* = 0.114 for the source × Cu-loading interaction.

For cycle-5 total passed charge (Figure 13b), two-way ANOVA yielded *p* = 0.749 for nanocellulose source, *p* = 0.156 for Cu loading, and *p* = 0.389 for their interaction. Analysis of the specimen-level Q^+^/|Q^−^| ratios yielded *p* = 0.0353 for Cu loading, *p* = 0.0615 for source, and *p* = 0.695 for their interaction. Mean total passed charge values were close between the two sources at 15 and 50 wt% Cu, while larger numerical differences occurred at 25 and 75 wt%.

### 3.3. Thermogravimetric Analysis of Cu-Modified Nanocellulose

**Figure 14:**
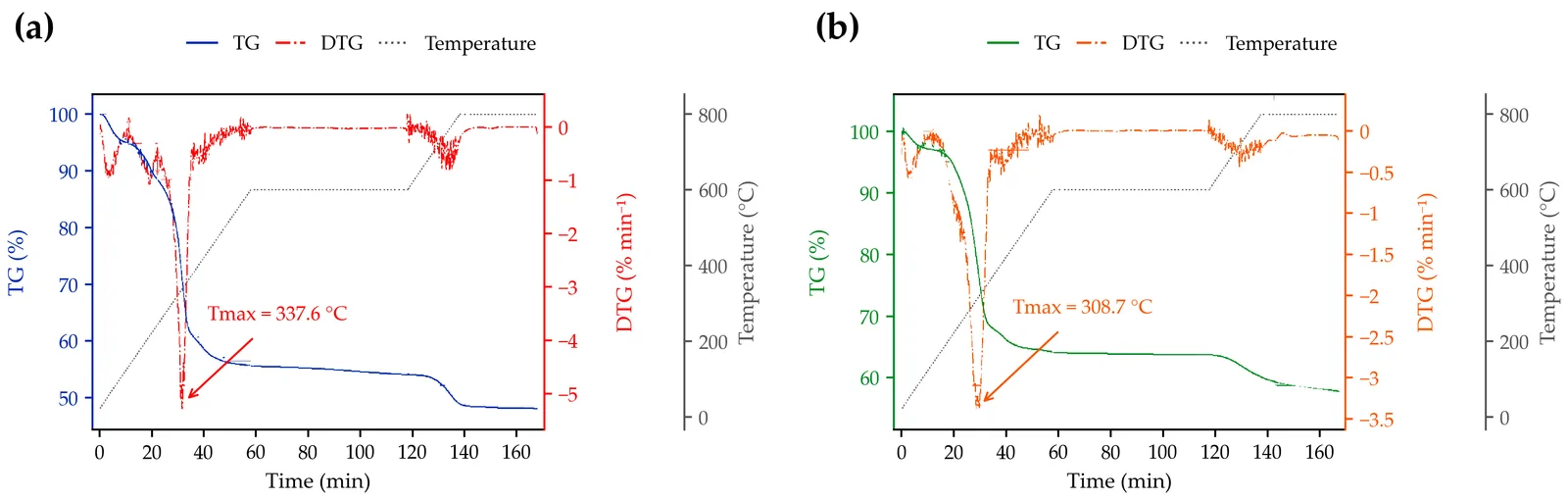
TG, DTG, and temperature–time profiles of (**a**) H-NC-Cu 50% and (**b**) C-NC-Cu 50%. Solid colored lines represent TG, dash-dotted colored lines represent DTG, and gray dotted lines show the recorded temperature program against the outer right-hand axis. The N_2_ flow indicated in the instrument output corresponds to protective-gas flow and was not used as the sample purge atmosphere.

Figure 14 presents the TG and DTG profiles of H-NC-Cu 50% and C-NC-Cu 50%, together with the recorded temperature–time program. The program comprises heating to 600 °C, an isothermal holding stage, subsequent heating to 800 °C, and a final holding stage. Displaying the complete profiles against time distinguishes the mass changes occurring during heating from those occurring during the isothermal stages.

Both formulations exhibited a pronounced mass-loss step accompanied by a principal DTG minimum. The instrument-labeled temperatures of maximum mass-loss rate were 337.6 °C for H-NC-Cu 50% and 308.7 °C for C-NC-Cu 50%. Further mass loss occurred during the later stages of the program, followed by a comparatively gradual change during the final hold. Table 4 reports final residues of 48.1 and 57.8 wt% for H-NC-Cu 50% and C-NC-Cu 50%, respectively. These values describe the total material remaining after thermal treatment. The accompanying CuO-equivalent Cu values are conditional stoichiometric conversions based on treating the residue as CuO; they do not independently establish its composition or the elemental Cu content.

### 3.4. X-Ray Diffraction Analysis of Cu-Modified Nanocellulose

**Figure 15:**
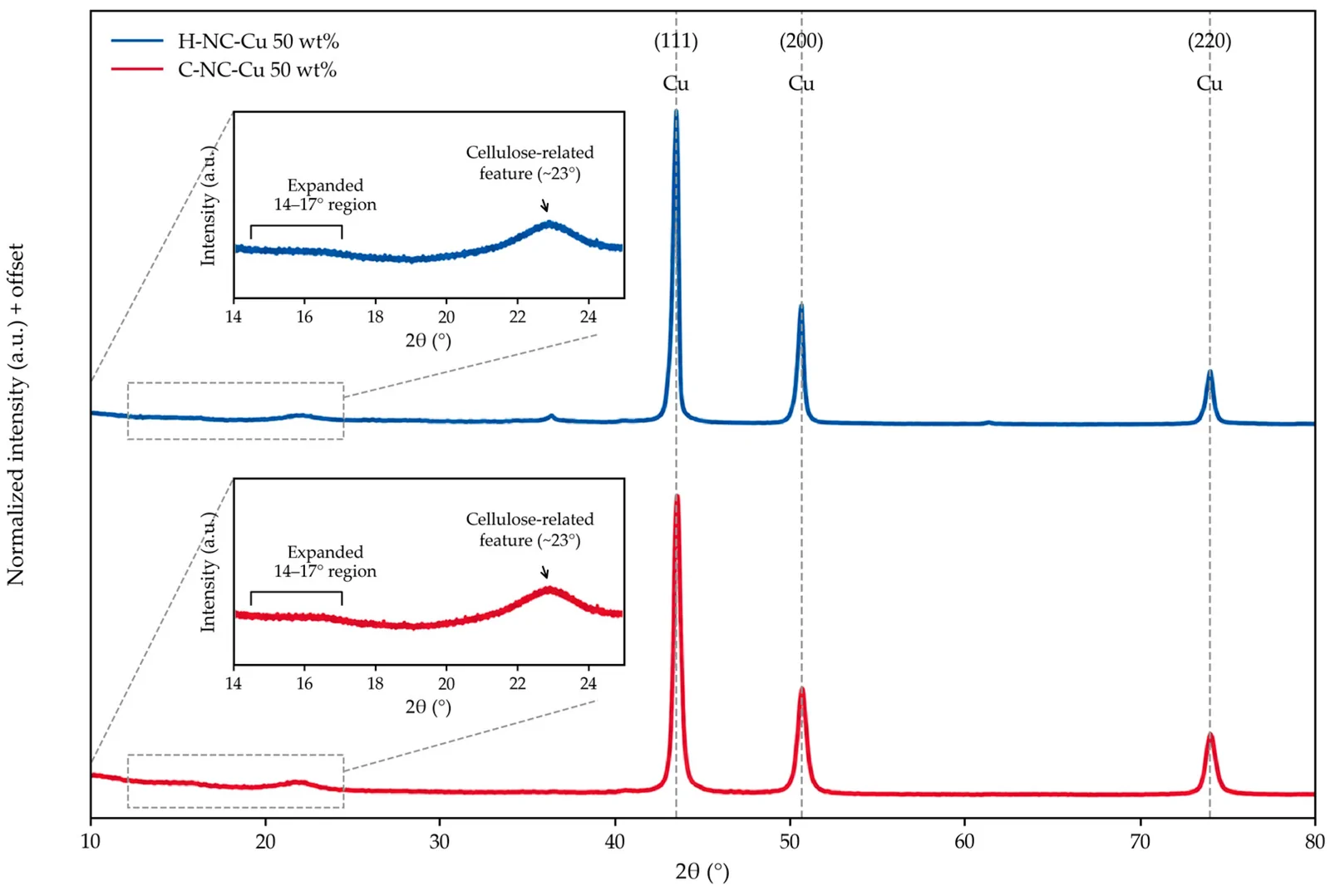
XRD patterns of H-NC-Cu 50 wt% and C-NC-Cu 50 wt%.

XRD patterns of H-NC-Cu 50% and C-NC-Cu 50% are presented in Figure 15. Both composites exhibit pronounced reflections at approximately 2θ = 43.2–43.4°, 50.3–50.5°, and 74.0–74.2°, assigned to the (111), (200), and (220) planes of face-centered cubic metallic Cu, respectively [25,34,35]. These characteristic reflections confirm the presence of crystalline metallic Cu in both nanocellulose matrices. The expanded low-angle regions reveal a weak broad feature near 22.5–23.0° and weaker features within 14–17°, consistent with cellulose-associated diffraction [36]. Thus, the patterns show prominent crystalline Cu reflections alongside the weaker diffraction contribution of the cellulose matrix. The patterns are normalized and vertically offset for visual comparison. The Cu peaks were substantially more intense than the cellulose-related features.

## 4. Discussion

### 4.1. Source-Dependent Optimization of Nanocellulose Extraction

The RSM results show that extraction cycle number and sonication time cannot be treated as interchangeable measures of processing severity. In hemp hurds, the effects of extraction cycle number depended on sonication time and differed between TCI and apparent fibril width. At 75 min sonication, increasing the cycle number from one to three increased TCI from 1.061 to a mean of 3.636, while the mean apparent fibril width increased from 76.7 to 97.74 nm. At 120 min sonication, the fourth-cycle sample exhibited a lower TCI and a smaller apparent fibril width than the selected three-cycle sample. This non-monotonic behavior is consistent with literature showing that repeated mechanical fibrillation can change both fibrillation efficiency and cellulose structural characteristics rather than producing a simple linear improvement with additional passes [3,9]. The result therefore supports the use of cycle number as an explicit process variable in nanocellulose optimization.

Corn cob showed a different processing window. The three-cycle/75 min condition provided a reproducible compromise between TCI and fibril size, whereas more severe treatment did not improve both responses simultaneously. Previous chemical–mechanical production of corn-cob nanocellulose has likewise emphasized the need to balance chemical purification with mechanical disintegration [4]. More broadly, the present source dependence agrees with the established observation that feedstock composition and native fiber architecture influence nanocellulose dimensions, structural organization, and fibrillation behavior [1,2,3].

RSM supported the selection of source-specific extraction conditions by considering TCI and apparent fibril width together. A linear model described hemp-hurd TCI, whereas quadratic models described hemp-hurd fibril width and both corn-cob responses. At the selected conditions, the predicted fibril widths were close to the experimental values for both sources, and the predicted corn-cob TCI also showed close numerical agreement with the measured response. The measured hemp-hurd TCI exceeded its predicted value, indicating that agreement differed between responses. The selected conditions were therefore assessed using numerical optimization together with experimental FTIR and SEM observations. Model comparisons and diagnostic statistics are provided in the Supplementary Materials. This combined use of modeling and experimental assessment is consistent with RSM-based nanocellulose extraction studies [17,19,20].

### 4.2. FTIR and SEM Evidence of Cellulose Enrichment and Fibrillation

The persistence of the principal cellulose bands after treatment, together with the strong reduction in the features near 1730, 1510, and 1240 cm^−1^, supports substantial removal of hemicellulose- and lignin-associated components while preserving the cellulose-rich framework [4,18]. The FTIR comparison therefore complements the RSM response by showing that the selected 3-120H and 3-75C materials are chemically enriched in cellulose relative to the untreated feedstocks. Because FTIR-derived TCI is a band-intensity ratio, its absolute magnitude should be interpreted as a relative structural-order index within the present datasets rather than as a direct crystallinity percentage [30].

SEM provided independent morphological confirmation of nanoscale fibrillation. At the same time, the spread of apparent feature sizes and the field-dependent morphology caution against interpreting any individual micrograph as a single uniform fibril diameter. The source-specific optimization therefore rests on the combined response of FTIR-derived structural order and SEM-derived size rather than on either technique alone.

### 4.3. Effect of Cu Incorporation on Electrochemical Response

The clearest functional effect of Cu modification was the large reduction in low-frequency impedance and the tens-of-fold increase in cycle-5 passed charge relative to bare nanocellulose. Conductive nanocellulose systems generally require incorporation of an electrically active phase because the cellulose framework itself is poorly conducting [23,24]. Cu-containing cellulose and nanocellulose systems have also been reported as functional hybrid materials [25,26], and impregnation of cellulose fibers with Cu colloids has been shown to produce electrically conductive cellulose-based materials [37]. In the present samples, the strongest separation between bare and Cu-modified materials occurred at low frequency, whereas the high-frequency Z′ values of the Cu-containing groups remained comparatively close. This pattern indicates that Cu incorporation altered the slower network/interfacial response much more strongly than the high-frequency resistance proxy. The overall behavior is also consistent with the broader use of nanoparticle-decorated nanocellulose as a platform for conductive and electrochemically active hybrid materials [38].

The loading dependence differed between the two biomass sources. Corn-cob-derived nanocellulose showed a progressive decrease in |Z| from 15 to 75 wt% nominal Cu, whereas the hemp-hurd series reached its lowest mean impedance in the 25–50 wt% range and increased again at 75 wt%. The passed-charge response was also non-monotonic: the largest mean response occurred in the 25–50 wt% region for hemp and at 50 wt% for corn cob. Thus, increasing nominal Cu loading beyond 50 wt% did not yield a universal improvement in the combined electrochemical metrics.

The 50 wt% formulation provided the most consistent compromise across the two sources, combining relatively low impedance with high passed charge. However, this should be treated as a comparative trend within the present dataset rather than as a statistically established global optimum. Several loading comparisons did not reach conventional statistical significance, and the Cu-modified groups were based on *n* = 3 independent preparations. The bare controls were measured once and should therefore remain descriptive references rather than the basis of inferential comparisons.

Forward and reverse scans provide information about the dependence of the current–potential response on scan direction and history [39,40]. In the present measurements, hysteresis was observed between the forward and reverse potential sweeps.

A physically meaningful equivalent-circuit model is necessary for interpreting fitted EIS parameters [41]. In the present analysis, the fitted resistances could not be assigned a validated charge-transfer interpretation. The EIS results were therefore discussed in terms of the directly measured impedance magnitude, phase response, and their variation with Cu loading.

The Lin-KK assessment complemented the comparison of the measured impedance magnitude and phase responses by providing information about the internal consistency of the spectra [32,33]. The remaining frequency-dependent residual deviations were considered when interpreting the EIS results, particularly at the lowest measured frequencies, and uniform KK consistency over the complete frequency range was not assumed. The assessment did not establish a specific physical or instrumental origin for these deviations. Detailed residual profiles and sensitivity checks are provided in the Supplementary Materials.

### 4.4. Biomass Matrix Effects and the Relationship Between Cu Retention and Performance

The cross-source comparison indicates that biomass origin modulates the effect of Cu loading rather than producing a simple overall ranking of the two feedstocks. The two sources were nearly identical at 15 wt% Cu and similar again at 50 wt%, while larger differences appeared at 25 and 75 wt%. This behavior is consistent with the broader understanding that nanocellulose source and processing history affect morphology, surface chemistry, and network organization, all of which can influence the connectivity and electroanalytical response of an incorporated conductive phase [1,3,24,42].

The thermal profiles complement the structural observations obtained by FTIR, SEM, and XRD. FTIR supports cellulose enrichment after extraction, SEM shows nanoscale fibrillation, and XRD identifies crystalline metallic Cu alongside weaker cellulose-associated diffraction features in the selected 50 wt% formulations. The TG and DTG profiles therefore describe the thermal behavior of composites containing a cellulose-rich matrix and a crystalline Cu component. The principal mass-loss-rate peak occurred at a higher temperature for H-NC-Cu 50% than for C-NC-Cu 50% under the recorded program. The thermal behavior of cellulose nanomaterials depends on their source and processing history, while heating conditions also influence decomposition pathways and char formation [43,44]. Copper-precursor content and processing conditions have likewise been reported to affect cellulose degradation in a cotton-based Cu nanoparticle synthesis system [34].

This difference distinguishes the principal decomposition events of the two formulations, without establishing a direct relationship between their FTIR-derived structural-order indices and thermal stability.

The corn-cob-derived 50 wt% formulation retained more final residue than the corresponding hemp-hurd-derived formulation (Table 4). This observation complements the detection of crystalline Cu by XRD, although residual mass and diffraction features provide different information about the composites. The residue can contain Cu-containing products and other nonvolatile constituents and therefore cannot be assigned entirely to elemental Cu. Considered alongside the electrochemical results, the thermal findings show that final residue alone does not determine impedance or passed-charge response. The combined measurements describe source-dependent structural, thermal, and electrochemical behavior.

### 4.5. Structural Confirmation by XRD and Integrated Interpretation

The reflections observed in both 50 wt% Cu-modified samples are consistent with crystalline face-centered cubic metallic Cu, as also reported for copper nanoparticles supported on nanocrystalline cellulose [25]. The weak broad feature near 23° is consistent with a contribution from the cellulose matrix. However, the cellulose-related features are insufficiently resolved in these composite patterns to establish the cellulose polymorph unambiguously. The structural interpretation therefore combines the qualitative XRD observations with the cellulose-related FTIR bands and SEM evidence of nanoscale fibrillation, without treating these measurements as equivalent determinations of crystallinity [36].

Together, the extraction, structural, electrochemical, and thermal measurements describe source- and loading-dependent responses. The electrochemical results do not show a uniform improvement with increasing nominal Cu loading. The 50 wt% corn-cob formulation exhibited the highest mean cycle-5 total passed charge, whereas the hemp-hurd formulations showed a different loading dependence. Accordingly, the 50 wt% samples provide a common composition for structural and thermal comparison, rather than establishing a universal optimum for both biomass sources.

## 5. Conclusions

Nanocellulose was obtained from hemp hurds and corn cob through a citric-acid-assisted chemical–mechanical route. RSM identified source-specific extraction conditions of three treatment cycles with 120 min sonication for hemp hurds and 75 min for corn cob. FTIR showed retention of the principal cellulose bands and reduced lignin- and hemicellulose-associated features, supporting cellulose enrichment after treatment. The FTIR-derived TCI, LOI, and HBI provided complementary relative spectral descriptors, while SEM confirmed nanoscale fibrillation. The selected hemp-hurd and corn-cob materials exhibited mean apparent fiber sizes of 83.3 and 100 nm, respectively.

Cu modification was investigated as a means of enhancing the electrochemical response of the extracted nanocellulose and exploring its potential for electrochemical applications. Cu incorporation at nominal loadings of 15–75 wt% produced substantially lower low-frequency impedance and greater passed charge than the corresponding bare nanocellulose references. The loading dependence differed between the two sources: the hemp-hurd series exhibited a non-monotonic impedance response, whereas the corn-cob series showed decreasing mean impedance with increasing nominal Cu loading and its highest mean cycle-5 total passed charge at 50 wt%. These observations identify composition-dependent electrochemical responses under the measurement conditions and support further investigation of the composites for electrochemical applications.

XRD confirmed crystalline face-centered cubic metallic Cu in both selected 50 wt% formulations, alongside weaker cellulose-associated diffraction features. Their thermal profiles showed principal temperatures of maximum mass-loss rate of 337.6 °C for H-NC-Cu 50% and 308.7 °C for C-NC-Cu 50%, together with different final residual masses. These complementary observations distinguish the structural, thermal, and electrochemical characteristics of the two formulations. Overall, the results support source-specific extraction and Cu modification as a route for producing agricultural-residue-derived nanocellulose composites with enhanced electrochemical response.

## Figures and Tables

**Table 1:** Selected RSM conditions and experimental confirmation.

Source	Selected Sample	Cycles	Sonication (min)	Predicted TCI	Predicted Size (nm)	Desirability	Observed Response
Hemp hurds	3-120H	3	120	1.632	90	0.894	TCI 5.735; size 83.3 nm
Corn cob	3-75C	3	75	2.06	94.3	0.713	TCI 2.242 ± 0.002 size 99.6 ± 2.1 nm

**Table 2:** Low-frequency EIS response of bare and Cu-modified nanocellulose.

Source	Cu Loading	N	|Z| at 0.1 Hz (kΩ)	−Phase at 0.1 Hz (°)
Hemp hurds	Bare	1	274.15	81.5
Hemp hurds	15 wt%	3	9.65 ± 0.92	76.2 ± 5.4
Hemp hurds	25 wt%	3	8.44 ± 1.27	59.3 ± 13.7
Hemp hurds	50 wt%	3	8.44 ± 0.63	63.0 ± 8.8
Hemp hurds	75 wt%	3	9.45 ± 0.29	69.0 ± 6.2
Corn cob	Bare	1	247.67	80.4
Corn cob	15 wt%	3	9.90 ± 0.81	77.7 ± 3.4
Corn cob	25 wt%	3	9.65 ± 0.44	75.8 ± 2.3
Corn cob	50 wt%	3	8.81 ± 1.02	61.0 ± 7.2
Corn cob	75 wt%	3	8.17 ± 0.84	69.3 ± 2.3

**Table 3:** Cycle-5 passed charge of bare and Cu-modified nanocellulose.

Source	Cu Loading	N	Q^+^ (mC)	|Q^−^| (mC)	Total Charge (mC)
Hemp hurds	Bare	1	0.0049	0.0055	0.0104
Hemp hurds	15 wt%	3	0.251 ± 0.019	0.248 ± 0.046	0.500 ± 0.047
Hemp hurds	25 wt%	3	0.461 ± 0.359	0.337 ± 0.169	0.798 ± 0.528
Hemp hurds	50 wt%	3	0.477 ± 0.134	0.299 ± 0.083	0.777 ± 0.208
Hemp hurds	75 wt%	3	0.284 ± 0.058	0.252 ± 0.025	0.536 ± 0.053
Corn cob	Bare	1	0.0054	0.0055	0.0109
Corn cob	15 wt%	3	0.254 ± 0.069	0.249 ± 0.031	0.503 ± 0.098
Corn cob	25 wt%	3	0.262 ± 0.043	0.246 ± 0.023	0.507 ± 0.066
Corn cob	50 wt%	3	0.450 ± 0.042	0.370 ± 0.090	0.821 ± 0.132
Corn cob	75 wt%	3	0.321 ± 0.065	0.343 ± 0.046	0.665 ± 0.111

**Table 4:** Final TGA residue at 800 °C and CuO-equivalent Cu content of Cu-modified nanocellulose.

Sample	Nominal Cu Loading (wt%)	Final Residue at 800 °C (wt%)	CuO-Equivalent Cu Content (wt%)
H-NC-Cu 15%	15	24.7	19.7
H-NC-Cu 25%	25	35.1	28.0
H-NC-Cu 50%	50	48.1	38.4
H-NC-Cu 75%	75	56.8	45.4
C-NC-Cu 15%	15	51.2	40.9
C-NC-Cu 25%	25	56.0	44.7
C-NC-Cu 50%	50	57.8	46.2
C-NC-Cu 75%	75	61.05	48.8

## Data Availability

The original contributions presented in this study are included in the article/Supplementary Materials. Further inquiries can be directed to the corresponding author.

## Supplementary Materials

The following supporting information can be downloaded at: https://www.mdpi.com/article/10.3390/polym18202547/s1, Table S1: Hemp-hurd experimental design and recorded responses; Table S2: Corn-cob experimental design and recorded responses; Table S3: Diagnostic statistics for the selected response models; Table S4: Selected software solutions and experimental comparisons; Table S5: Model-form comparisons displayed in the supplied fit-summary reports; Table S6: Hemp-hurd coefficients in terms of the tabulated actual factors; Table S7: Hemp-hurd selected-model ANOVA; Table S8: Corn-cob size coefficients in terms of coded factors, as displayed by Stat-Ease; Table S9: Corn-cob size ANOVA transcribed from the supplied software output; Table S10: Corrected absorbance heights and additional FTIR indices; Table S11: Lin-KK fitting settings and numerical checks; Table S12: Residual statistics for all individual recordings; Table S13: Model-order sensitivity for both regression modes; Table S14: Resistance sensitivity for the nine boundary-limited Cu-modified spectra; Figure S1: FTIR baseline and TCI band-height visualization for 1-30H, 1-30C, 1-75H, 1-75C, 1-120H, 1-120C, 1-140H, and 1-140C; Figure S2: FTIR baseline and TCI band-height visualization for 3-11H, 3-11C, 3-75H, 3-75C, 3-120H, 3-120C, 3-140H, and 3-140C; Figure S3: FTIR baseline and TCI band-height visualization for 4-30H, 4-30C, 4-120H, 4-120C, 5-75H, and 5-75C; Figure S4: Additional SEM images of 1-30H, 1-30C, 1-75H, 1-75C, 1-120H, 1-120C, 3-140H, and 3-140C; Figure S5: Additional SEM images of 3-11H, 3-11C, 4-120H, 4-120C, 4-30H, 4-30C, 5-75H, and 5-75C; Figure S6: Modulus-normalized Lin-KK residuals for hemp-hurd spectra using complex linear regression; Figure S7: Modulus-normalized Lin-KK residuals for corn-cob spectra using complex linear regression; Figure S8: Modulus-normalized Lin-KK residuals for hemp-hurd spectra using real-component regression; Figure S9: Modulus-normalized Lin-KK residuals for corn-cob spectra using real-component regression. References [29,31,32,33,45] are cited in Supplementary Materials File.
