# Supplementary material for: Response-Surface-Guided Extraction and Copper Modification of Nanocellulose from Corn Cob and Hemp Hurds: Source- and Loading-Dependent Structural and Electrochemical Properties
Source: Polymers (Basel). 2026 Oct 16;18(20):2547. doi: 10.3390/polym18202547 (PMC13708852; doi:10.3390/polym18202547)
Supplement: Supplementary file 1 [file polymers-18-02547-s001.zip › polymers-4631127-supplementary.pdf]

Supporting Information

Response-Surface-Guided Extraction and Copper Modification of Nanocellulose from Corn Cob and Hemp Hurds: Source- and Loading-Dependent Structural and Electrochemical Properties

Manuscript polymers-4631127

This document provides the RSM design matrices and available model outputs, FTIR baseline and band-height analyses, additional SEM images, and the numerical Lin-KK assessment supporting the main manuscript. H and C identify hemp-hurd- and corn-cob-derived materials, respectively.

S1 Response surface methodology

The 13-run design matrices below reproduce the supplied current Stat-Ease experiment tables. TCI is dimensionless and apparent fiber size is expressed in nm. The response values used for RSM are retained as reported; they are not replaced by newly calculated ratios from the FTIR exports. Five records occur at three cycles and 75 min in each design.

Table S1. Hemp-hurd experimental design and recorded responses.

Run	Standard order	Cycles	Sonication (min)	TCI	Apparent size (nm)
1	6	5	75	3.315	97.0
2	2	4	30	1.330	130.0
3	13	3	75	3.732	100.0
4	12	3	75	3.597	99.0
5	5	1	75	1.061	76.7
6	11	3	75	3.667	95.0
7	10	3	75	3.543	96.7
8	3	1	120	1.136	86.7
9	1	1	30	1.172	143.0
10	4	4	120	1.098	68.7
11	7	3	11	7.239	140.0
12	8	3	140	0.851	110.0
13	9	3	75	3.641	98.0

S1 Response surface methodology continued

Table S2. Corn-cob experimental design and recorded responses.

Run	Standard order	Cycles	Sonication (min)	TCI	Apparent size (nm)
1	5	1	75	1.106	113.3
2	3	1	120	1.156	113.3
3	11	3	75	2.243	102.0
4	10	3	75	2.240	100.0
5	8	3	140	1.123	146.0
6	12	3	75	2.241	97.0
7	1	1	30	1.071	130.0
8	7	3	11	3.768	160.0
9	4	4	120	0.540	66.7
10	13	3	75	2.242	101.0
11	6	5	75	0.530	80.0
12	9	3	75	2.245	98.0
13	2	4	30	1.292	83.3

The FTIR dataset comprises 11 distinct processing conditions for each source. These spectra and the 13-run RSM matrices are identified separately: repeated center-condition response records do not imply that corresponding replicate FTIR spectra were supplied.

The selected samples used for subsequent Cu modification were 3-120H and 3-75C. Their experimental comparison is reported with the numerical solutions in Table S4.

S1 Model diagnostics and numerical solutions

Table S3. Diagnostic statistics for the selected response models.

**Source and response**	**Model**	**Model p**	**R^2**	**Adjusted R^2**	**Predicted R^2**	**Lack of fit p**
Hemp TCI	Linear	0.1150	0.3512	0.2214	-0.3084	<0.0001
Hemp size	Quadratic	0.0279	0.7833	0.6286	-0.7755	0.0003
Corn TCI	Quadratic	Not shown	0.8407†	0.7269	−0.2690	<0.0001
Corn size	Quadratic	0.0518	0.7364	0.5481	−1.1215	0.0001

R^2 describes the proportion of response variation represented by the fitted model. Adjusted R^2 accounts for the number of fitted terms. Predicted R^2 is a PRESS-based diagnostic and can be negative. The reported diagnostic values are retained alongside the experimental comparisons; desirability is not an independent measure of predictive accuracy.

Table S4. Selected software solutions and experimental comparisons.

**Source**	**Cycles**	**Sonication (min)**	**Predicted TCI**	**Predicted size (nm)**	**Desirability**	**Measured TCI**	**Measured size (nm)**
Hemp hurds	3	120	1.63249	89.9576	0.894	5.735	83.3
Corn cob	3	75	2.06402	94.3332	0.713	2.242	100

Software solutions are transcribed from the supplied solution reports. Experimental selected-sample values are those reported in the revised manuscript. The hemp-hurd TCI prediction differs from the measured value; agreement in the selected processing conditions does not imply equal agreement for every response.

S1 Available model comparisons

Table S5. Model-form comparisons displayed in the supplied fit-summary reports.

Source and response	Model	Sequential p	Lack of fit p	Adjusted R^2	Predicted R^2	Status
Hemp TCI	Linear	0.1150	<0.0001	0.2214	−0.3084	Selected
Hemp TCI	2FI	0.6462	<0.0001	0.1560	−1.1279	Compared
Hemp TCI	Quadratic	0.3445	<0.0001	0.1997	−3.0109	Compared
Hemp TCI	Cubic	<0.0001	—	0.9985	—	Aliased
Hemp size	Linear	0.0248	0.0002	0.4271	−0.0127	Compared
Hemp size	2FI	0.8335	0.0001	0.3666	−0.5470	Compared
Hemp size	Quadratic	0.0641	0.0003	0.6286	−0.7755	Selected
Hemp size	Cubic	0.0003	—	0.9924	—	Aliased
Corn TCI	Linear	0.2480	<0.0001	0.0921	−0.5229	Compared
Corn TCI	2FI	0.4812	<0.0001	0.0483	−1.3906	Compared
Corn TCI	Quadratic	0.0053	<0.0001	0.7269	−0.2690	Selected
Corn TCI	Cubic	<0.0001	—	1.0000	—	Aliased

S1 Hemp-hurd coefficients and analysis of variance

Table S6. Hemp-hurd coefficients in terms of the tabulated actual factors.

Term	TCI linear coefficient	Size quadratic coefficient
Intercept	3.40535447	168.21126149
A	0.43902391	2.44462232
B	-0.02574948	-1.57538740
AB	—	0.02657812
A^2	—	-0.85881460
B^2	—	0.00705696

A is the number of cycles and B is sonication time in min. The linear equation contains the intercept and A and B terms; the quadratic equation also contains AB, A^2 and B^2. These coefficients were independently calculated by ordinary least squares from Table S1.

Table S7. Hemp-hurd selected-model ANOVA.

Response	Source	Sum of squares	df	Mean square	F	p
TCI	Model	14.29423	2	7.14712	2.7062	0.1150
TCI	Residual	26.41056	10	2.64106	—	—
TCI	Lack of fit	26.39018	6	4.39836	863.6097	<0.0001
TCI	Pure error	0.02037	4	0.00509	—	—
TCI	Corrected total	40.70479	12	—	—	—
Size	Model	4755.14653	5	951.02931	5.0619	0.0279
Size	Residual	1315.16424	7	187.88061	—	—
Size	Lack of fit	1299.81224	3	433.27075	112.8897	0.0003
Size	Pure error	15.35200	4	3.83800	—	—
Size	Corrected total	6070.31077	12	—	—	—

Values were recalculated from Table S1. Pure error is calculated from the five repeated center-condition response records. Dashes indicate that the statistic is not applicable.

S1 Corn-cob size model output

Table S8. Corn-cob size coefficients in terms of coded factors, as displayed by Stat-Ease.

Term	Coefficient	Standard error	95% CI lower	95% CI upper	VIF
Intercept	105.07	8.18	85.73	124.40	—
A	−14.00	6.51	−29.39	1.38	1.02
B	−6.85	6.32	−21.78	8.09	1.05
AB	0.5174	8.52	−19.63	20.66	1.05
A^2	−11.52	7.79	−29.94	6.90	1.03
B^2	19.36	6.48	4.05	34.68	1.01

Table S9. Corn-cob size ANOVA transcribed from the supplied software output.

Source	Sum of squares	df	Mean square	F	p
Model	5997.47	5	1199.49	3.91	0.0518
A	1421.14	1	1421.14	4.63	0.0683
B	360.44	1	360.44	1.18	0.3142
AB	1.13	1	1.13	0.0037	0.9533
A^2	670.45	1	670.45	2.19	0.1828
B^2	2742.25	1	2742.25	8.94	0.0202
Residual	2146.67	7	306.67	—	—
Lack of fit	2129.47	3	709.82	165.08	0.0001
Pure error	17.20	4	4.30	—	—
Corrected total	8144.15	12	—	—	—

S2 FTIR baseline and TCI band heights

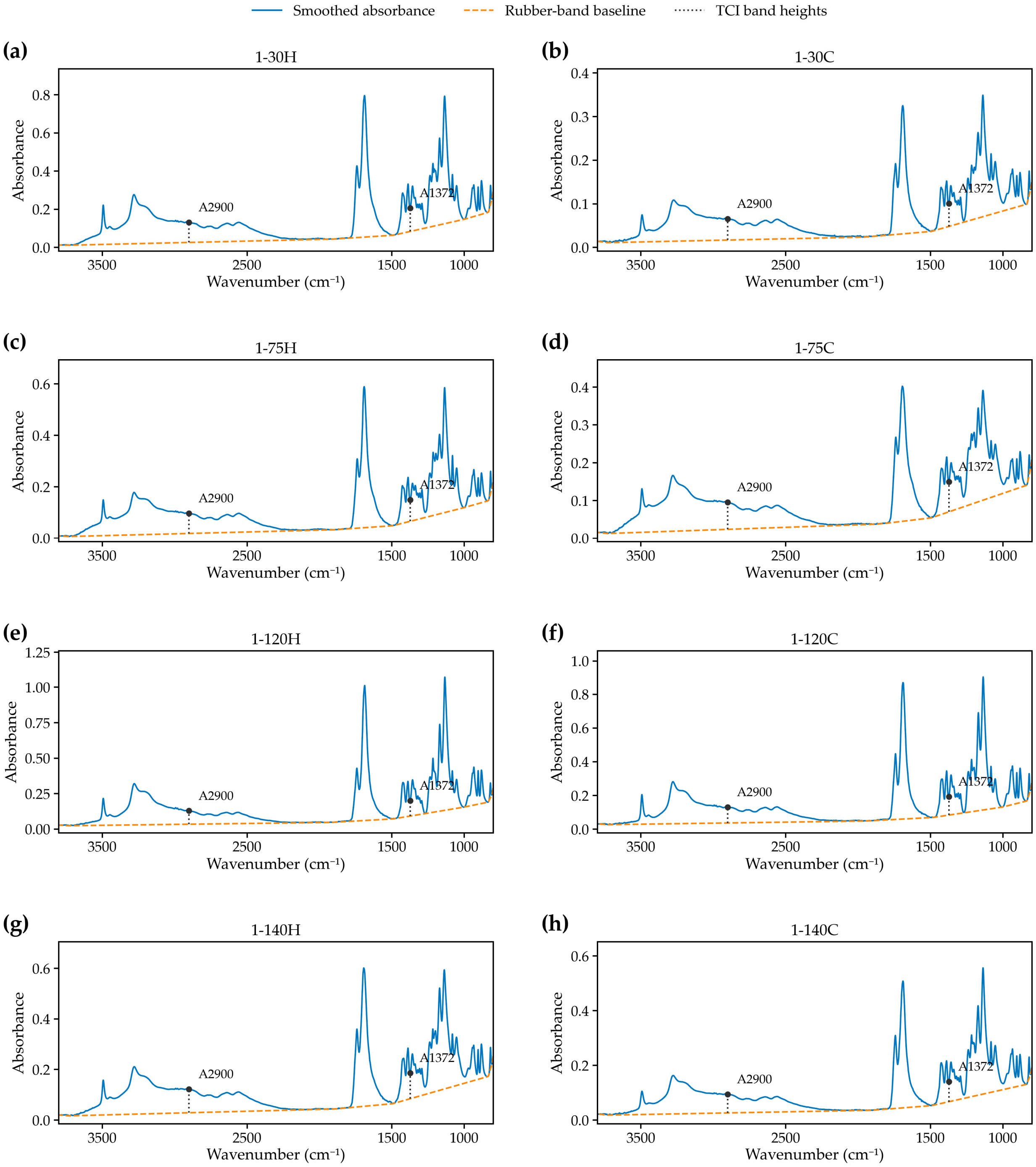

Figure S1. FTIR baseline and TCI band-height visualization: (a) 1-30H; (b) 1-30C; (c) 1-75H; (d) 1-75C; (e) 1-120H; (f) 1-120C; (g) 1-140H; (h) 1-140C. Blue lines show smoothed absorbance, orange dashed lines show the lower-envelope rubber-band baseline, and gray dotted segments mark the corrected absorbance heights at 1372 and 2900 cm^{-1}. Spectra are plotted before normalization or vertical offsets.

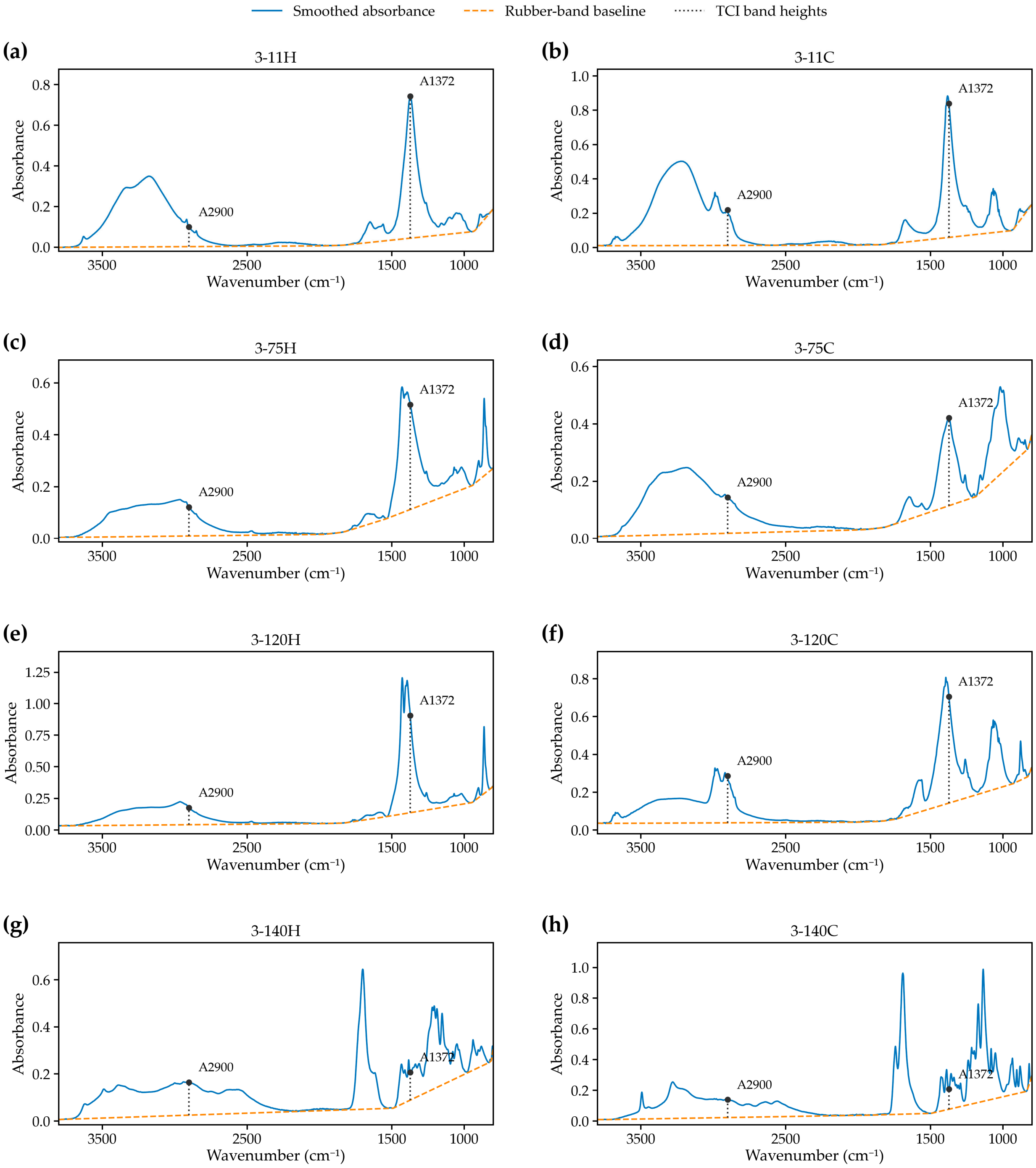

Figure S2. FTIR baseline and TCI band-height visualization: (a) 3-11H; (b) 3-11C; (c) 3-75H; (d) 3-75C; (e) 3-120H; (f) 3-120C; (g) 3-140H; (h) 3-140C. Blue lines show smoothed absorbance, orange dashed lines show the lower-envelope rubber-band baseline, and gray dotted segments mark the corrected absorbance heights at 1372 and 2900 cm^{-1}. Spectra are plotted before normalization or vertical offsets.

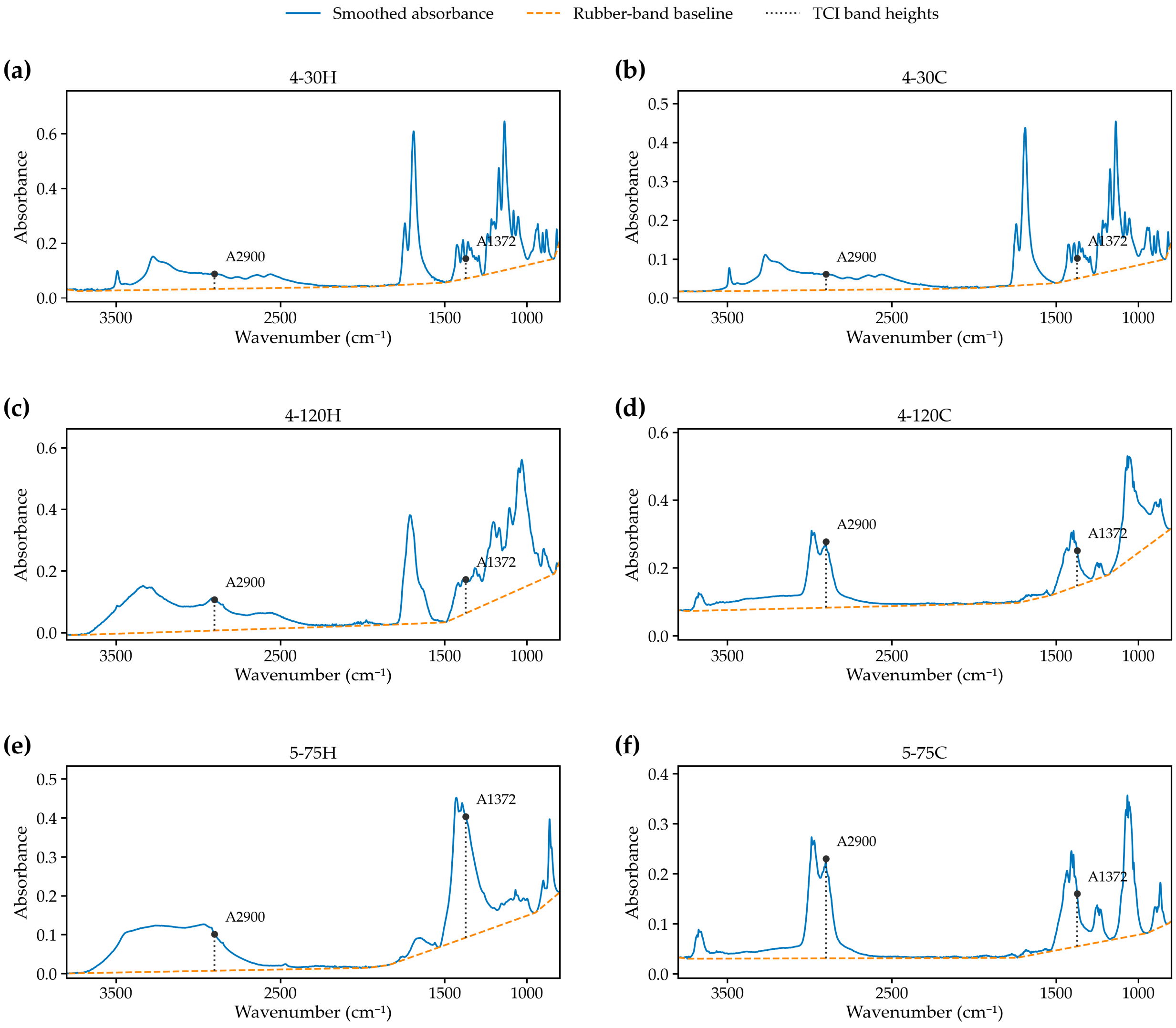

Figure S3. FTIR baseline and TCI band-height visualization: (a) 4-30H; (b) 4-30C; (c) 4-120H; (d) 4-120C; (e) 5-75H; (f) 5-75C. Blue lines show smoothed absorbance, orange dashed lines show the lower-envelope rubber-band baseline, and gray dotted segments mark the corrected absorbance heights at 1372 and 2900 cm^{-1}. Spectra are plotted before normalization or vertical offsets.

S3 Additional FTIR index calculations

All 22 spectra were processed using the procedure specified for TCI. Percentage transmittance T was converted to absorbance as $A = -\log_{10}(T/100)$. Savitzky–Golay smoothing used an 11-point window and a third-order polynomial. A lower-envelope rubber-band baseline over 800–3800 cm^{-1} was constructed as a piecewise linear lower convex hull of the smoothed absorbance spectrum. The baseline-corrected height at each specified band was obtained by linear interpolation. TCI is defined as the absorbance-height ratio A_{1372}/A_{2900} [1]. Figures S1–S3 show the baseline and these two band heights.

LOI = A_{1430}/A_{897} and HBI = A_{3340}/A_{1330}, where the subscripts denote wavenumber in cm^{-1} and A denotes corrected absorbance height [2]. Calculations were performed before normalization or vertical offsets. The ratios are dimensionless empirical spectral descriptors, rather than absolute crystallinity percentages. OH-related interpretation also depends on moisture [3]. Each value represents one supplied spectrum; replicate standard deviations were not calculated.

Table S10. Corrected absorbance heights and additional FTIR indices.

Sample	A_{1430}	A_{897}	LOI	A_{3340}	A_{1330}	HBI
1-30H	0.18165	0.04886	3.718	0.10976	0.12570	0.873
1-30C	0.08733	0.03421	2.553	0.03965	0.05543	0.715
1-75H	0.13303	0.04476	2.972	0.08207	0.10547	0.778
1-75C	0.10571	0.03502	3.019	0.08325	0.08121	1.025
1-120H	0.19890	0.09154	2.173	0.10265	0.12238	0.839
1-120C	0.18362	0.05426	3.384	0.07759	0.10895	0.712
1-140H	0.14283	0.04046	3.530	0.09106	0.09760	0.933
1-140C	0.13323	0.04775	2.790	0.06043	0.08372	0.722
3-11H	0.28516	0.03382	8.431	0.29111	0.41791	0.697
3-11C	0.24731	0.04188	5.905	0.41104	0.38079	1.079
3-75H	0.48352	0.07104	6.807	0.10766	0.26246	0.410
3-75C	0.20082	0.06254	3.211	0.21777	0.19212	1.133
3-120H	1.04704	0.05933	17.649	0.12116	0.30573	0.396
3-120C	0.44914	0.03563	12.607	0.12692	0.26904	0.472
3-140H	0.16610	0.06978	2.380	0.13034	0.13038	1.000
3-140C	0.20342	0.06686	3.042	0.10739	0.15755	0.682
4-30H	0.11683	0.04616	2.531	0.04407	0.07920	0.557
4-30C	0.08286	0.02768	2.994	0.02922	0.05202	0.562
4-120H	0.09760	0.09644	1.012	0.15030	0.10925	1.376
4-120C	0.11787	0.11131	1.059	0.03205	0.02838	1.129
5-75H	0.36827	0.06057	6.080	0.11328	0.21465	0.528
5-75C	0.15008	0.04430	3.388	0.01308	0.03601	0.363

Band heights are reported to five decimal places and ratios to three decimal places. Ratios were calculated using unrounded values. The original TCI responses used for RSM remain those in Tables S1 and S2. Applying the displayed baseline to the revised exports gives TCI values of 5.735 for 3-120H and 2.242 for 3-75C. The selected 3-120H and 3-75C spectra yield LOI/HBI values of 17.649/0.396 and 3.211/1.133, respectively.

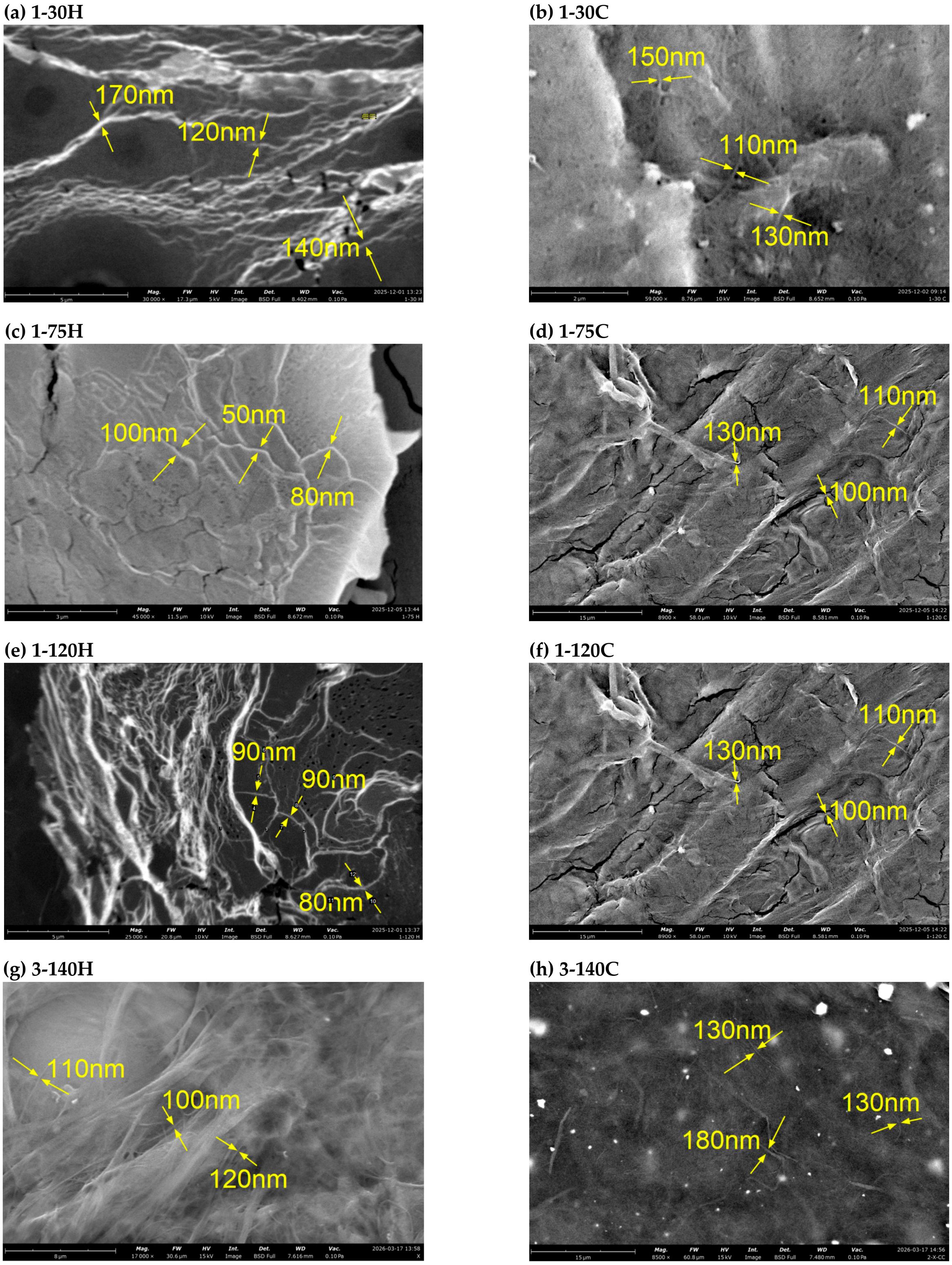

Figure S4. Additional SEM images: (a) 1-30H; (b) 1-30C; (c) 1-75H; (d) 1-75C; (e) 1-120H; (f) 1-120C; (g) 3-140H; (h) 3-140C. Original scale bars and measurement annotations are retained. H and C denote hemp-hurd- and corn-cob-derived samples, respectively. The selected 3-120H and 3-75C images are presented in the main manuscript and are not repeated here. Source-image check: panels (d) and (f) contain the identical image assigned to 1-75C and 1-120C in the supplied Word file; the sample assignment requires verification against the original SEM records.

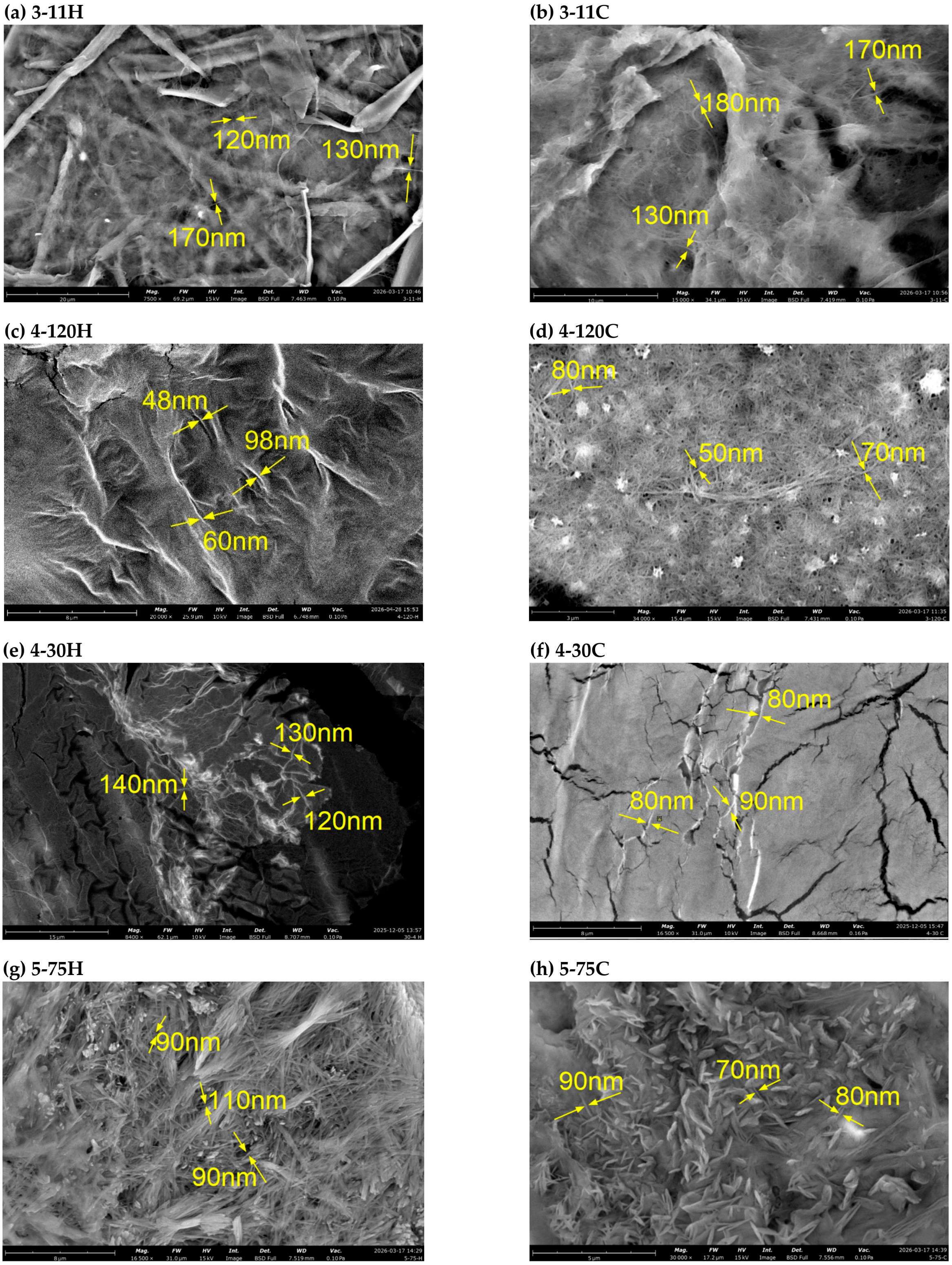

Figure S5. Additional SEM images: (a) 3-11H; (b) 3-11C; (c) 4-120H; (d) 4-120C; (e) 4-30H; (f) 4-30C; (g) 5-75H; (h) 5-75C. Original scale bars and measurement annotations are retained. H and C denote hemp-hurd- and corn-cob-derived samples, respectively. The selected 3-120H and 3-75C images are presented in the main manuscript and are not repeated here.

S4 Linear Kramers–Kronig assessment

The 26 spectra comprise 24 Cu-modified specimens, with three independently prepared specimens per source and loading, and one recording for each bare source. All 61 measured frequency points from 0.1 Hz to 100 kHz were retained for each spectrum. EIS measurements used 0.1 M $KHCO_3$ electrolyte, a 10 mV AC perturbation and an applied DC potential of 0 V. The KK assessment was performed separately for every spectrum [4, 5].

The measured complex impedance was reconstructed from the exported real and negative imaginary columns. Exported modulus and phase agreed with the values calculated from this reconstruction. C50-1, C50-2 and C50-3 represent independent specimens. C75 specimen 2 uses the retained recording C75-2-2; the alternate technical acquisition C75-2-1 was excluded.

A series resistance and a sum of RC terms with logarithmically spaced fixed time constants were fitted using modulus-weighted complex linear least squares. Series capacitance and inductance terms were included. The time-constant interval was $1/(2\pi f_{max})$ to $1/(2\pi f_{min})$. The model order increased until the resistor-mass parameter μ reached or fell below 0.85, subject to a maximum of 50 RC terms. This cutoff controls model order and is not a criterion for spectrum validity [5].

Table S11. Lin-KK fitting settings and numerical checks.

Item	Setting or check
Primary regression	Modulus-weighted complex linear least squares
Frequency points	61 per spectrum; all points retained
Frequency interval	0.1 Hz to 100 kHz
Fixed time constants	Logarithmically spaced from $1/(2\pi f_{max})$ to $1/(2\pi f_{min})$
Series terms	Resistance, inductance and capacitance
Order cutoff / maximum	μ = 0.85 / maximum M = 50
Sensitivity cutoffs	μ = 0.75 and 0.95
Additional regression	Real-component regression
Implementation check	impedance.py 1.7.1 with identical settings
Independent solver	QR least-squares verification of complex fits

At each frequency, the real and imaginary residuals are the measured minus fitted components divided by the measured impedance modulus and multiplied by 100. The complex RMS residual is the square root of the mean sum of the squared real and imaginary percentage residuals over the 61 points. The maximum component residual is the largest absolute real or imaginary residual at any measured frequency.

S4 Individual residual statistics

Table S12. Residual statistics for all individual recordings.

Recording	M	μ	Complex fit RMS (%)	Complex fit max (%)	Real fit RMS (%)	Real fit max (%)
H	17	0.8490	0.348	1.701	0.507	2.580
H15-1	16	0.8231	0.726	3.492	0.852	4.610
H15-2	16	0.8494	1.068	2.561	1.258	3.424
H15-3	17	0.8230	0.457	1.915	0.654	2.947
H25-1	17	0.8224	0.690	2.896	0.773	3.298
H25-2	20	0.8477	0.942	2.785	1.163	4.303
H25-3	16	0.8196	0.841	4.398	1.027	5.924
H50-1	17	0.8144	1.057	4.508	1.381	6.440
H50-2	18	0.8302	1.402	5.354	1.983	8.572
H50-3	16	0.8205	0.826	4.219	1.059	5.796
H75-1	16	0.8479	0.933	3.110	1.100	4.204
H75-2	16	0.8219	1.076	5.331	1.226	6.561
H75-3	16	0.8468	1.346	4.193	1.561	6.102
C	17	0.7984	1.071	2.626	1.626	5.054
C15-1	17	0.8239	0.700	1.770	0.794	2.188
C15-2	16	0.8132	0.657	3.418	0.794	4.487
C15-3	17	0.7955	0.404	1.801	0.498	2.484
C25-1	16	0.8071	1.459	4.105	1.761	5.650
C25-2	16	0.8393	0.541	2.647	0.627	3.438
C25-3	17	0.8258	0.736	2.039	0.840	2.627
C50-1	16	0.8328	0.805	3.552	0.932	4.525
C50-2	17	0.8253	1.186	5.138	1.577	7.515
C50-3	16	0.8224	1.493	6.827	2.085	10.181
C75-1	20	0.8179	1.019	1.859	1.175	2.509
C75-2	16	0.8443	1.034	5.077	1.366	7.169
C75-3	17	0.8498	0.631	3.395	0.824	4.729

M and μ correspond to the primary complex fit. “Max” denotes the maximum absolute component residual, not the maximum complex modulus of the residual. “Real fit RMS” remains the complex RMS statistic evaluated after real-component regression. Values are fit diagnostics rather than replicate SDs. C75-2 denotes the retained C75-2-2 recording.

Complex-fit RMS residuals ranged from 0.35% to 1.49%. Frequency-dependent deviations were generally more pronounced near the low-frequency end. The largest absolute component residual was 6.83% at 0.1 Hz for C50-3. Real-component regression gave complex RMS residuals of 0.50–2.09% and a maximum component residual of 10.18% for the same recording. No universal residual threshold or binary pass/fail label was assigned.

S4 Regression verification and model-order sensitivity

Table S13. Model-order sensitivity for both regression modes.

Recording	Complex c = 0.75	Complex c = 0.85	Complex c = 0.95	Real c = 0.75	Real c = 0.85	Real c = 0.95
H	19 / 0.321	17 / 0.348	15 / 0.384	23 / 0.490	20 / 0.507	17 / 0.533
H15-1	18 / 0.659	16 / 0.726	13 / 0.885	23 / 0.787	19 / 0.852	13 / 1.008
H15-2	18 / 1.042	16 / 1.068	14 / 1.090	21 / 1.247	19 / 1.258	15 / 1.274
H15-3	19 / 0.429	17 / 0.457	15 / 0.488	22 / 0.641	20 / 0.654	17 / 0.678
H25-1	19 / 0.583	17 / 0.690	15 / 0.808	20 / 0.696	18 / 0.773	16 / 0.877
H25-2	22 / 0.917	20 / 0.942	18 / 0.963	25 / 1.144	23 / 1.163	22 / 1.172
H25-3	18 / 0.741	16 / 0.841	13 / 0.991	20 / 0.919	17 / 1.027	14 / 1.154
H50-1	19 / 0.983	17 / 1.057	14 / 1.184	21 / 1.344	19 / 1.381	16 / 1.462
H50-2	20 / 1.354	18 / 1.402	15 / 1.463	23 / 1.963	22 / 1.983	21 / 1.998
H50-3	18 / 0.756	16 / 0.826	14 / 0.910	20 / 1.013	18 / 1.059	15 / 1.133
H75-1	18 / 0.894	16 / 0.933	14 / 0.978	21 / 1.074	19 / 1.100	16 / 1.137
H75-2	18 / 0.995	16 / 1.076	13 / 1.220	21 / 1.170	17 / 1.226	14 / 1.341
H75-3	19 / 1.267	16 / 1.346	13 / 1.459	27 / 1.528	24 / 1.561	17 / 1.564
C	18 / 1.059	17 / 1.071	14 / 1.120	22 / 1.620	20 / 1.626	17 / 1.651
C15-1	19 / 0.684	17 / 0.700	15 / 0.725	22 / 0.778	18 / 0.794	16 / 0.813
C15-2	18 / 0.588	16 / 0.657	13 / 0.771	20 / 0.753	18 / 0.794	15 / 0.872
C15-3	18 / 0.380	17 / 0.404	15 / 0.458	19 / 0.478	18 / 0.498	16 / 0.548
C25-1	18 / 1.414	16 / 1.459	13 / 1.545	20 / 1.732	18 / 1.761	14 / 1.831
C25-2	18 / 0.482	16 / 0.541	14 / 0.601	20 / 0.590	18 / 0.627	15 / 0.699
C25-3	19 / 0.713	17 / 0.736	15 / 0.762	21 / 0.825	19 / 0.840	16 / 0.868
C50-1	18 / 0.729	16 / 0.805	13 / 0.957	20 / 0.841	17 / 0.932	14 / 1.065
C50-2	19 / 1.089	17 / 1.186	15 / 1.288	21 / 1.544	20 / 1.577	17 / 1.675
C50-3	18 / 1.393	16 / 1.493	14 / 1.598	22 / 2.055	20 / 2.085	17 / 2.146
C75-1	22 / 1.013	20 / 1.019	18 / 1.028	24 / 1.176	22 / 1.175	19 / 1.184
C75-2	18 / 0.956	16 / 1.034	14 / 1.112	21 / 1.328	19 / 1.366	17 / 1.408
C75-3	19 / 0.576	17 / 0.631	16 / 0.661	23 / 0.792	20 / 0.824	18 / 0.855

Each entry gives M / complex RMS residual (%); c denotes the μ cutoff controlling model order. The full frequency-resolved residuals and sensitivity statistics are provided as numerical data accompanying this document.

For C50-3, the maximum component residual remained at 0.1 Hz when the cutoff changed to 0.75 or 0.95: 6.10–7.45% for complex fitting and 9.96–10.58% for real-component fitting. The sensitivity analysis characterizes the observed deviation without assigning its physical origin.

Complex fits computed by column-scaled singular-value decomposition were reproduced using an independent QR solver and checked against impedance.py 1.7.1. The largest modulus-normalized fitted-impedance difference against the package was approximately 1.42×10^{-11}. An exactly representable synthetic RC spectrum was recovered to numerical precision; an intentional single-point perturbation produced a detectable residual. The NumPy namespace was supplied to the package evaluator for compatibility with NumPy 2 scalar representations, without changes to the fitting algorithm.

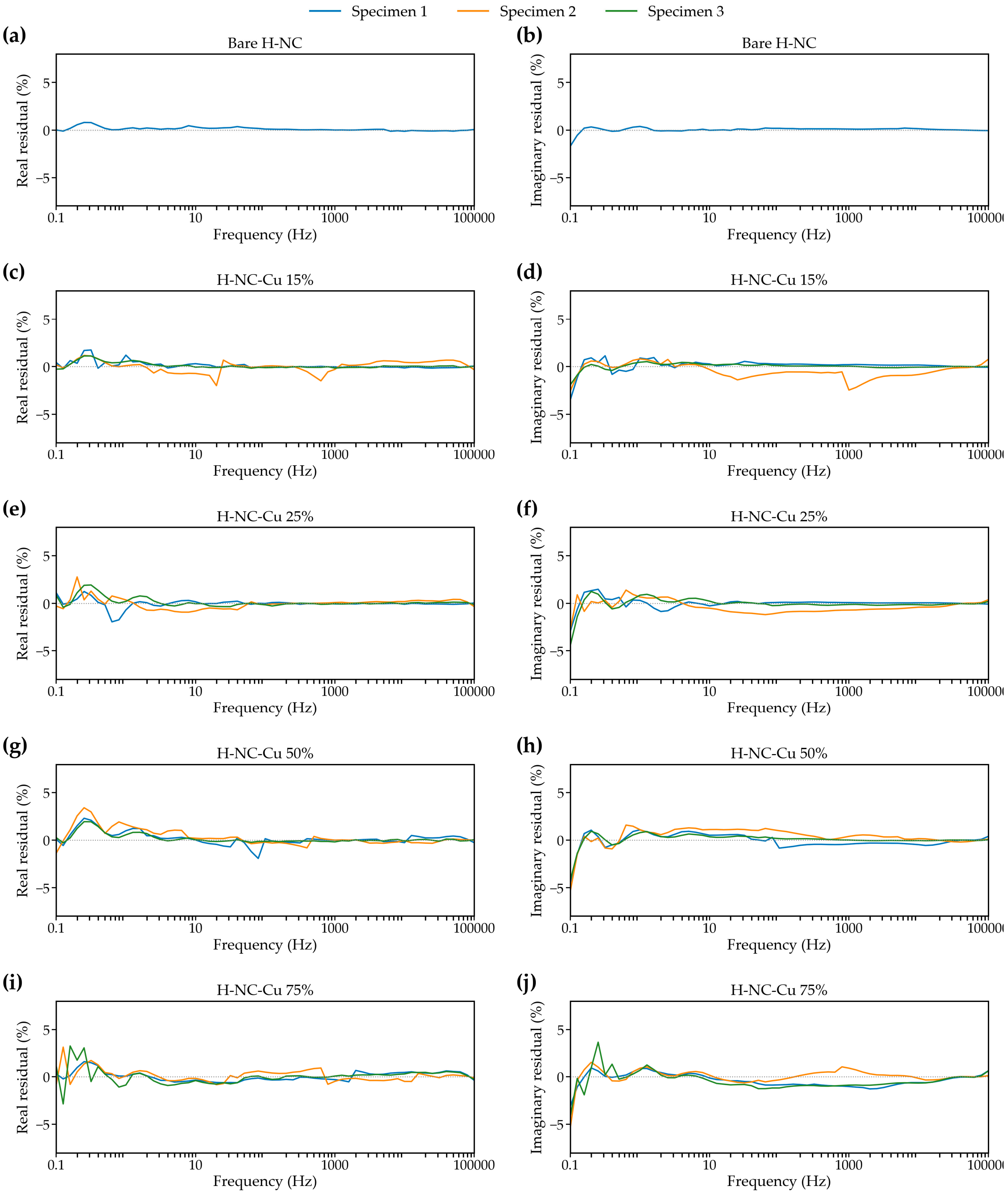

Figure S6. Modulus-normalized Lin-KK residuals for hemp-hurd spectra using complex linear regression. The left and right columns show real and imaginary residuals, respectively. Rows correspond to bare NC and nominal Cu loadings of 15, 25, 50, and 75 wt%. Colored lines represent the individual independent specimens; each bare reference has one recording. All 61 frequency points are retained. The zero line is a visual reference and not a pass/fail threshold.

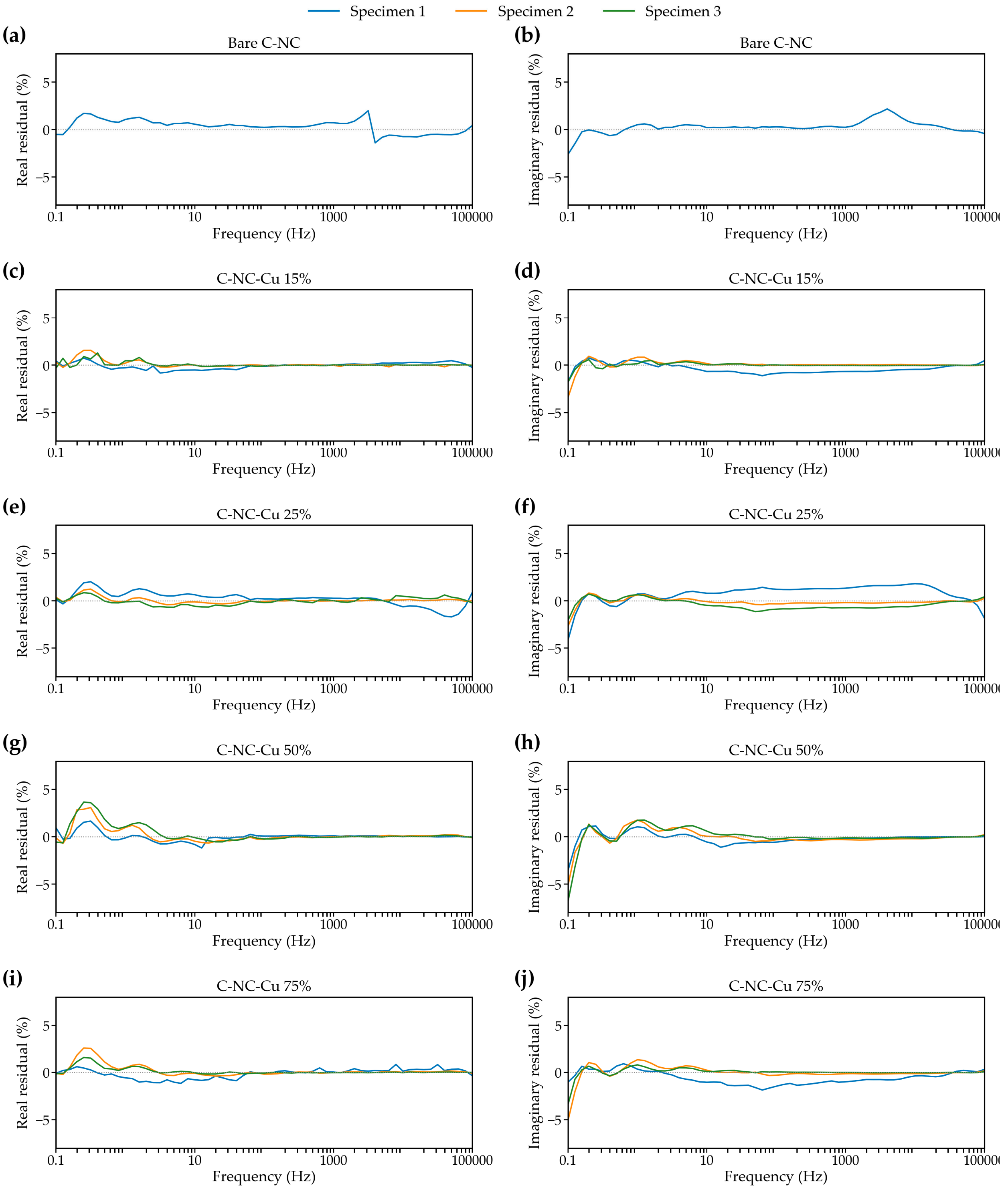

Figure S7. Modulus-normalized Lin-KK residuals for corn-cob spectra using complex linear regression. The left and right columns show real and imaginary residuals, respectively. Rows correspond to bare NC and nominal Cu loadings of 15, 25, 50, and 75 wt%. Colored lines represent the individual independent specimens; each bare reference has one recording. All 61 frequency points are retained. The zero line is a visual reference and not a pass/fail threshold.

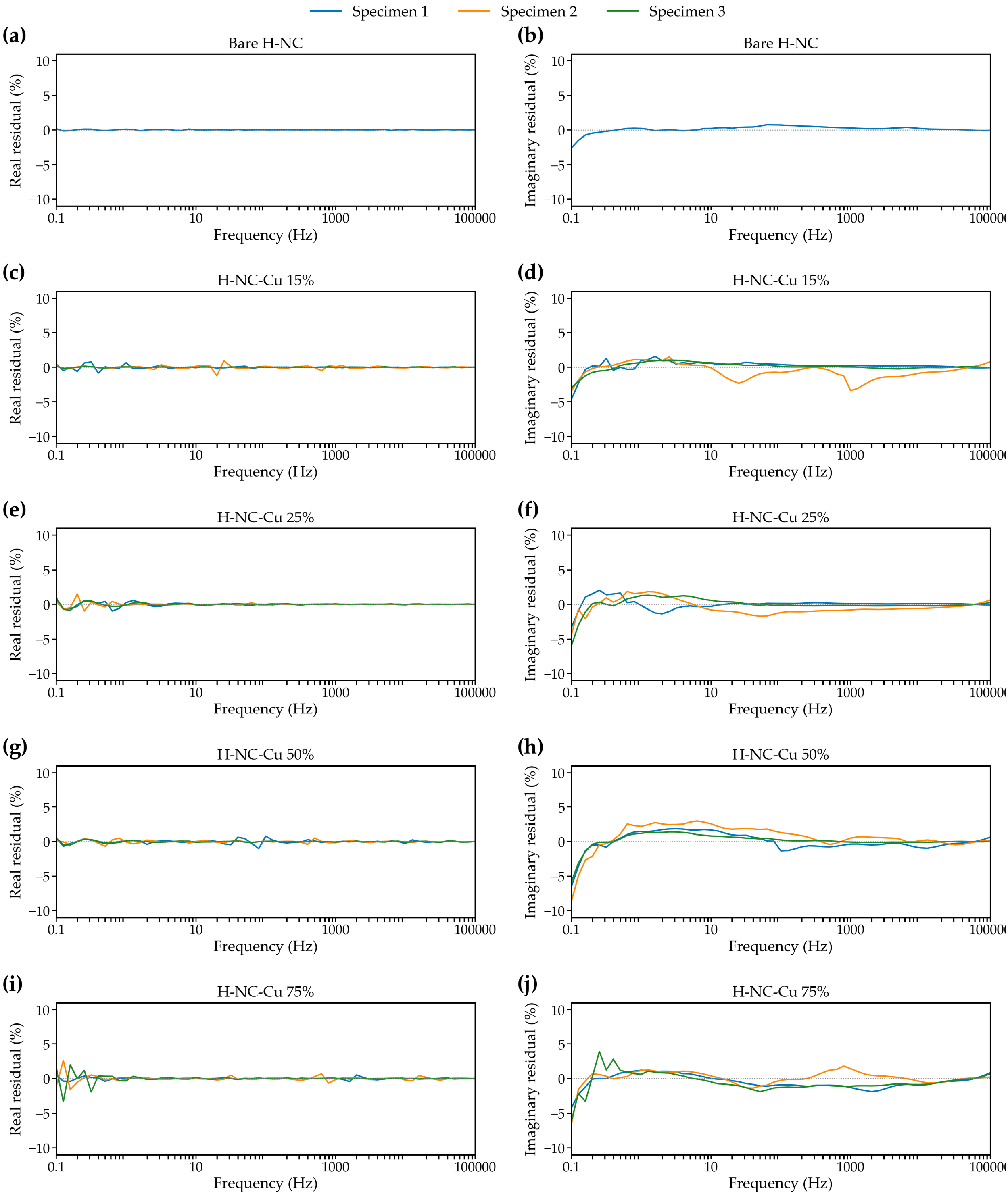

Figure S8. Modulus-normalized Lin-KK residuals for hemp-hurd spectra using real-component regression. The left and right columns show real and imaginary residuals, respectively. Rows correspond to bare NC and nominal Cu loadings of 15, 25, 50, and 75 wt%. Colored lines represent the individual independent specimens; each bare reference has one recording. All 61 frequency points are retained. The zero line is a visual reference and not a pass/fail threshold.

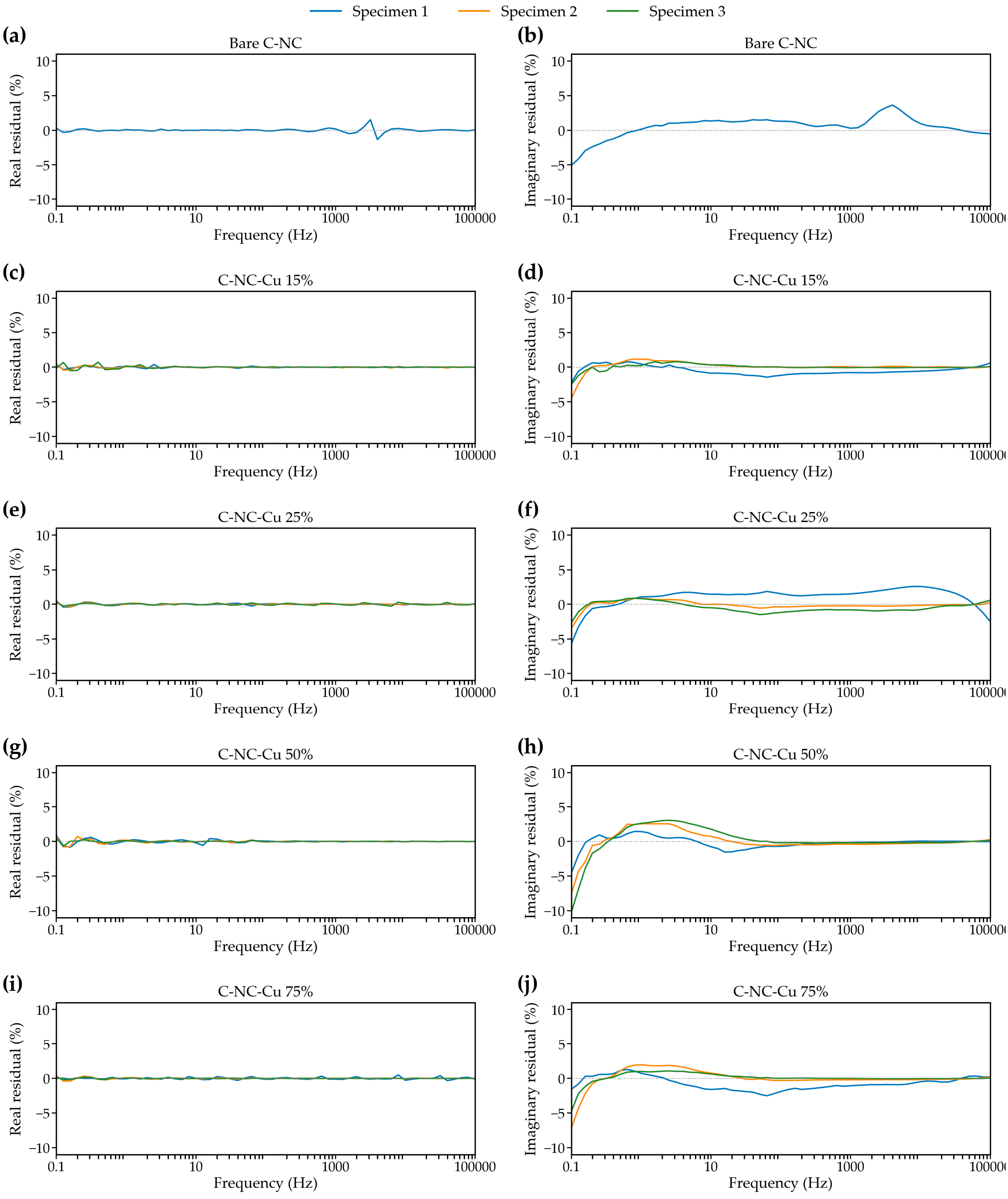

Figure S9. Modulus-normalized Lin-KK residuals for corn-cob spectra using real-component regression. The left and right columns show real and imaginary residuals, respectively. Rows correspond to bare NC and nominal Cu loadings of 15, 25, 50, and 75 wt%. Colored lines represent the individual independent specimens; each bare reference has one recording. All 61 frequency points are retained. The zero line is a visual reference and not a pass/fail threshold.

S5 Equivalent-circuit resistance sensitivity

Equivalent-circuit fitting was examined separately from the Lin-KK consistency assessment. For the exploratory circuit comprising Rs in series with a parallel R1–CPE1 branch, nine of the 24 Cu-modified spectra reached the imposed R1 upper bound of 10^{12} Ω. For these spectra, R1 was fixed successively at 10^{8}, 10^{1}[illegible]and 10^{12} Ω while the remaining parameters were refitted. These calculations examine parameter sensitivity and do not establish a physical charge-transfer resistance.

Table S14. Resistance sensitivity for the nine boundary-limited Cu-modified spectra.

**Recording**	**RMS (%)**<br>**R1 = 10^{8} Ω**	**RMS (%)**<br>**R1 = 10^{10} Ω**	**RMS (%)**<br>**R1 = 10^{12} Ω**	**RMS spread**<br>**(percentage points)**
C15-1	16.929269	16.928750	16.928745	0.000523888
C15-2	18.985430	18.984944	18.984939	0.000490936
C15-3	9.401348	9.401209	9.401208	0.000140238
C25-2	11.386065	11.385900	11.385899	0.000166041
C25-3	12.499271	12.498988	12.498985	0.000286109
C50-1	18.810000	18.809538	18.809534	0.000466418
C50-2	16.391014	16.390903	16.390902	0.000112884
C75-1	15.548793	15.548420	15.548416	0.000377324
C25-1	14.181495	14.181414	14.181414	0.000081051

The maximum spread was 0.000523888 percentage points. Circuit-fit residuals in this table are distinct from Lin-KK residuals. R1 is an exploratory circuit parameter and is not assigned to R_{ct}.

Adding a further series CPE yielded finite fitted resistances in exploratory fits, but finite parameter values and lower residuals alone did not establish a charge-transfer interpretation. Consequently, physically validated R_{ct} values were not reported from these models. Lin-KK measurement-model parameters likewise were not interpreted as R_{ct} or double-layer capacitance.

References

1. Nelson, M.L. and R.T. O'Connor, *Relation of certain infrared bands to cellulose crystallinity and crystal lattice type. Part II. A new infrared ratio for estimation of crystallinity in celluloses I and II.* Journal of Applied Polymer Science, 1964. **8**(3): p. 1325-1341. https://doi.org/10.1002/app.1964.070080323.
2. Hashemzehi, M., et al., *Enzyme-Enhanced Manufacturing of Cationized Dialdehyde Cellulose.* Biomacromolecules, 2025. **26**(9): p. 5581-5590. https://doi.org/10.1021/acs.biomac.4c01819.
3. Cichosz, S. and A. Masek, *IR study on cellulose with the varied moisture contents: Insight into the supramolecular structure.* Materials, 2020. **13**(20): p. 4573. https://doi.org/10.3390/ma13204573.
4. Boukamp, B.A., *A Linear Kronig-Kramers Transform Test for Immittance Data Validation.* Journal of The Electrochemical Society, 1995. **142**(6): p. 1885-1894. https://doi.org/10.1149/1.2044210.
5. Schönleber, M., D. Klotz, and E. Ivers-Tiffée, *A Method for Improving the Robustness of linear Kramers-Kronig Validity Tests.* Electrochimica Acta, 2014. **131**: p. 20-27. https://doi.org/10.1016/j.electacta.2014.01.034.

Numerical files accompanying this document

The numerical files include the transcribed RSM matrices, processed FTIR spectra and unrounded LOI/HBI results, individual measured EIS spectra, frequency-resolved measured/fitted impedances and residuals, model-order sensitivity results, and the verified KK reproduction scripts. The figure files are supplied separately as 600-dpi PNG and PDF. Original SEM annotations and scale bars are retained; source-image resolution is not increased by a higher export-dpi setting.
